# USP11 deubiquitinates E-cadherin and maintains the luminal fate of mammary tumor cells to suppress breast cancer

**DOI:** 10.1016/j.jbc.2024.107768

**Published:** 2024-09-11

**Authors:** Tao Qian, Feng Bai, Shiwen Zhang, Yuping Xu, Yuchan Wang, Shuping Yuan, Xiong Liu, Yaru Du, Bin Peng, Wei-Guo Zhu, Xingzhi Xu, Xin-Hai Pei

**Affiliations:** 1 Guangdong Provincial Key Laboratory of Regional Immunity and Diseases, International Cancer Center, Marshall Laboratory of Biomedical Engineering, The First Affiliated Hospital, Shenzhen University Medical School, Shenzhen University General Hospital, Shenzhen University, Shenzhen, Guangdong, China; 2 Department of Pathology, Shenzhen University Medical School, Shenzhen University, Shenzhen, Guangdong, China; 3 Gansu Dian Medical Laboratory, Lanzhou, China; 4 Guangdong Key Laboratory for Genome Stability & Disease Prevention and International Cancer Center, Shenzhen University Medical School, Shenzhen University, Shenzhen, Guangdong, China; 5 Department of Biochemistry and Molecular Biology, International Cancer Center, Shenzhen University Medical School, Shenzhen University, Shenzhen, Guangdong, China; 6 Department of Anatomy and Histology, Shenzhen University Medical School, Shenzhen University, Shenzhen, Guangdong, China

**Keywords:** USP11, deubiquitination, E-cadherin, breast cancer, luminal fate

## Abstract

Basal-like breast cancer may originate from luminal epithelial or cancerous cells. Inadequately repaired DNA damage impairs luminal differentiation and promotes aberrant luminal to basal trans-differentiation in mammary epithelial cells (MECs). Ubiquitin-specific peptidase 11 (USP11), a deubiquitinase, plays a critical role in DNA damage repair. The role of USP11 in controlling mammary cell differentiation and tumorigenesis remains poorly understood. We generated Usp11 knockout mice and breast cancer cell lines expressing wild-type (WT) and mutant forms of USP11. By using these mutant mice, cell lines, and human USP11-deficient and -proficient breast cancer tissues, we tested how USP11 controls mammary cell fate. We generated Usp11 knock-out mice and found that deletion of Usp11 reduced the expression of E-cadherin and promoted DNA damage in MECs. Overexpression of WT USP11, but not a deubiquitinase-inactive mutant form of USP11, promoted luminal differentiation, enhanced DNA damage repair, and suppressed tumorigenesis in mice. Mechanistically, we found that USP11 enhanced the protein expression of E-cadherin dependent on its deubiquitinase activity and that USP11 deubiquitinated E-cadherin at K738. We discovered that USP11 is bound to E-cadherin through its C-terminal region. In human breast cancers, expression of USP11 was positively correlated with that of E-cadherin, and high USP11 predicted better recurrence-free survival. Our findings provide compelling genetic and biochemical evidence that USP11 not only promotes DNA damage repair but also deubiquitinates E-cadherin and maintains the luminal feature of mammary tumor cells, thereby suppressing luminal breast cancer.

Breast cancer has been the most frequently diagnosed type of cancer since 2020, and it is also the second-leading cause of death in women (1). Breast cancer comprises of, among others, two main subtypes: estrogen receptor (ER)-positive luminal and ER-negative basal-like. Basal-like breast cancers (BLBCs) are highly invasive, poorly differentiated, and contain several distinct cell types such as cells that express luminal and basal biomarkers (2). We and others have demonstrated that some of the BLBCs originate from luminal epithelial or cancerous cells (3, 4, 5, 6, 7). Luminal breast cancers account for more than two-thirds of all diagnosed cases of breast cancer. In comparison with BLBCs, most luminal breast tumors are relatively well-differentiated and have a better prognosis due to their limited/marginal invasiveness, surgical resectability, and responsiveness to targeted hormone therapy (8, 9, 10). Due to the targeted therapy-induced drug resistance and yet unknown mechanisms, more than half of the luminal cancers relapse or lose their luminal and epithelial features, resulting in poorly differentiated cancers with enhanced invasiveness and metastasis (11). As such, the discovery of mechanisms controlling luminal features in breast cancer not only advances our understanding of the development and progression of luminal and basal-like cancers but also provides opportunities for developing new therapeutic strategies.

The integrity of the mammalian genome is under constant assault from external factors such as UV radiation and internal causes such as errors in DNA replication. Unrepaired or improperly repaired DNA damage results in chromosomal rearrangement and, ultimately, cancer, particularly hormone-related cancers such as breast cancer (12). We and others have shown that loss of Brca1, a gene associated with DNA damage repair and genomic stability maintenance, promotes luminal to basal and luminal to mesenchymal differentiation in mammary tumorigenesis and progression (3, 4, 5, 6, 13). More recently, it has been found that inadequate DNA damage repair induced by depletion of BRCA1 or its interacting proteins, including FANCD2, BRG1, NUMB, and HES1, impairs luminal differentiation and promotes aberrant luminal to basal trans-differentiation in immortalized MECs (14, 15). However, deficiency of BRCA2, a BRCA1-interacting protein that also functions in DNA damage repair, does not induce luminal-to-basal differentiation (14). The mechanisms associated with BRCA1-interacting proteins that modulate DNA damage repair and luminal cell differentiation in breast cancer development remain unclear.

The cell-to-cell adhesion molecule E-cadherin plays a central role in promoting and sustaining the polarization and differentiation status of epithelial cells (16). Loss of expression of E-cadherin (encoded by *CDH1*) impairs luminal and epithelial differentiation and induces an epithelial-to-mesenchymal transition (EMT) process, promoting invasion and metastasis in breast cancer (17, 18). CDH1 is regulated by several transcription factors, including GATA3 and most EMT-inducing transcription factors (EMT-TFs) (19, 20, 21, 22, 23). Although it has been reported that Hakai and MDM2 function as ubiquitin E3 ligases promoting the degradation of E-cadherin (24, 25), how E-cadherin is de-ubiquitinated remains elusive.

The deubiquitinase USP11 belongs to the deubiquitinating enzyme family, protecting ubiquitinated substrates from ubiquitin-mediated degradation by the removal of polyubiqutin chains (26). USP11 is a BRCA1-interacting protein and plays a critical role in DNA damage repair (27, 28). In addition to its function of being recruited to DNA break sites to directly participate in DNA damage repair, USP11 is also indirectly involved in repair through deubiquitination of some other DNA damage repair proteins including p53, γH2AX, p21, and H2BK120 (29, 30). The role of USP11 in regulating tumorigenesis and luminal or epithelial cell differentiation is quite contradictory. USP11 may promote EMT *in vitro* by deubiquitination of TGFBR2 and SNAIL, while it may also maintain luminal or epithelial cell features through stabilization of PTEN and positive regulation of ERα transcriptional activity (29, 30). It has been reported that, on the one hand, USP11 promotes cell proliferation and tumorigenesis through stabilizing its substrates cIAP2, NONO, NF90, E2F1, and cytoplasmic p21, but on the other hand, USP11 suppresses tumorigenesis by deubiquitinating its substrates PML, Mgl-1, PTEN, ARID1A, and nuclear p21 (29, 30). Regarding the function of USP11 in breast cancer, it, also confusingly, promotes breast cancer cell proliferation and tumorigenesis by stabilizing XIAP and cytoplasmic p21, and inhibits breast cancer cell proliferation through deubiquitination of PTEN (29, 30). Notably, other than a finding that loss of one allele of Usp11 in male mice destabilizes PTEN to promote mouse embryonic fibroblast (MEF) and prostate epithelial cell proliferation and tumorigenesis (31), all the findings on the role of USP11 in tumorigenesis and cell differentiation were achieved from cell culture-based systems. Considering that USP11 is an X chromosome-linked gene, it is important to uncover the function and mechanisms of USP11 in controlling female breast cancer development and progression *in vivo*.

In this study, we investigated the effect of USP11 in regulating mammary cell differentiation by using Usp11 knockout mice*,* breast cancer cell lines, and human breast cancer samples. We identified E-cadherin, a key protein in controlling luminal and epithelial cell differentiation, as a substrate of the USP11 deubiquitinase. We demonstrated that USP11 maintains the luminal fate of mammary tumor cells, thereby suppressing mammary tumorigenesis. USP11 is, therefore, a tumor suppressor in luminal breast cancer.

## Results

### Targeted deletion of the mouse Usp11 gene promotes DNA damage in MECs

**Figure 1:**
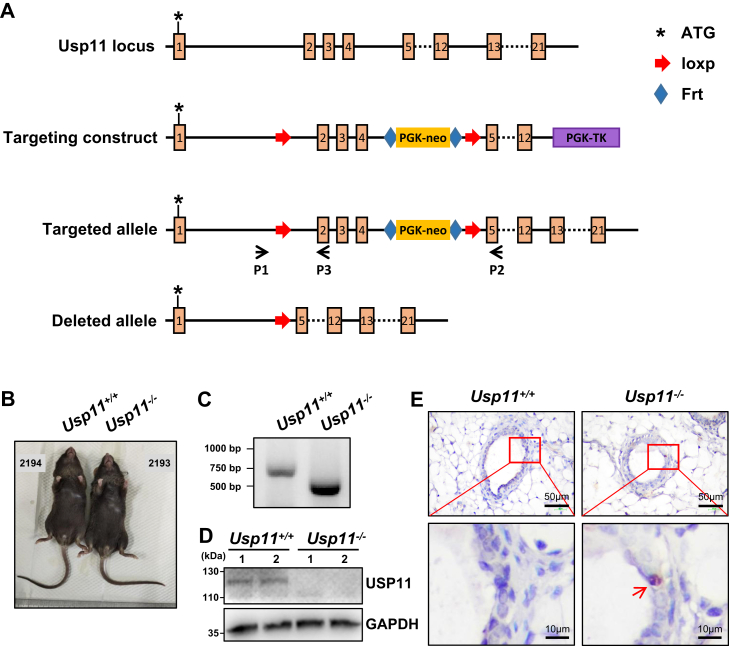
**Generation and characterization of Usp11 knock-out mice.***A*, schematic diagram of targeted deletion of exons 2 to 4 in the Usp11 gene in mice. *B*, representative gross appearance of 8-week-old *Usp11*^*+/+*^ and *Usp11*^*−/−*^ female mice. *C*, PCR analysis of genomic DNA from *Usp11*^*+/+*^ and *Usp11*^*−/−*^ mice. Wild-type band, 601 bp; null band, 464 bp. *D*, Western blotting analysis of the expression of USP11 in primary *Usp11*^*+/+*^ and *Usp11*^*−/−*^ MECs. GAPDH was used as an internal control. *E*, representative IHC staining of γH2AX in mammary glands from 5-week-old *Usp11*^*+/+*^ and *Usp11*^*−/−*^ mice.

To investigate the function of Usp11 in normal and tumor development *in vivo*, we generated Usp11 knock-out (*Usp11*^*−/−*^) mice in the BL/6 background (Fig. 1*A*; see details in “Materials and methods”). We confirmed the deletion of exons two to four by PCR and the lack of Usp11 protein expression in *Usp11*^*−/−*^ mice by Western blotting (Fig. 1, *C* and *D*). *Usp11*^*−/−*^ mice were developmentally normal, showing an indistinguishable gross appearance and comparable body weight to WT mice (Figs. 1*B* and S1*A*). Both male and female *Usp11*^*−/−*^ mice were fertile. These results are consistent with the findings described in other mice harboring different Usp11 knockout alleles (31, 32, 33).

To explore the impact of Usp11 deletion on DNA damage repair in MECs, we examined the expression of γH2AX, a marker for DNA double-strand breaks. We found that, on average, a positive MEC was detected in every 15 to 20 *Usp11*^*−/−*^ glands, while γH2AX-positive cells were rarely seen in *Usp11*^*+/+*^ glands (Fig. 1*E*). Due to the limitation of the study, we were unable to further quantify these results. In line with the finding that the deficiency of Usp11 induces defects in DNA repair (27, 28, 34, 35), these results indicate that Usp11 knockout induces DNA damage in mouse MECs.

### Usp11 positively regulates the expression of E-cadherin in mammary epithelial and tumor cells

**Figure 2:**
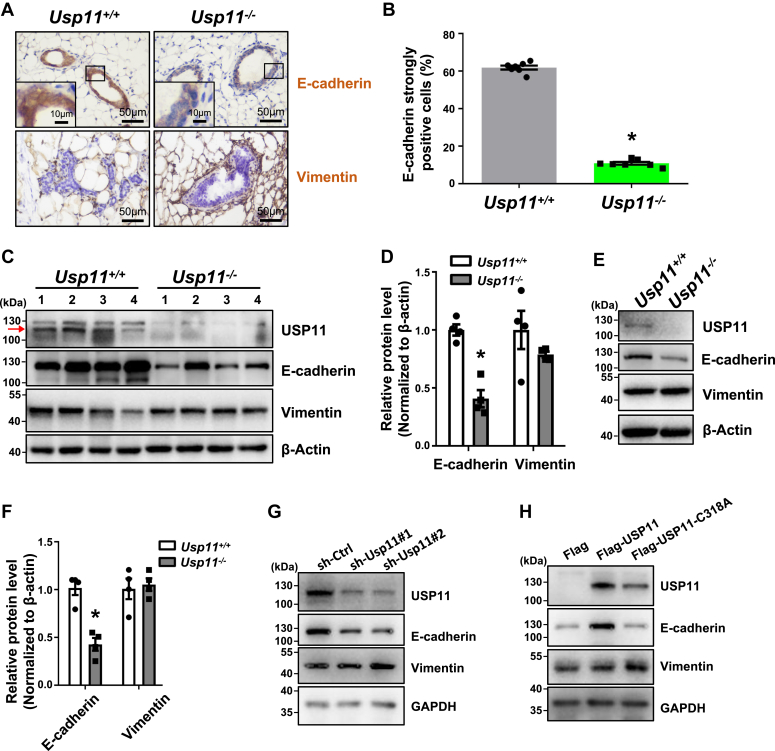
**USP11 regulates the expression of E-cadherin *in vivo* and *in vitro*.***A*, representative IHC staining of E-cadherin and Vimentin in mammary glands from 5-week-old *Usp11*^*+/+*^ and *Usp11*^*−/−*^ mice. Note the drastically reduced expression of E-cadherin in *Usp11*^*−/−*^ MECs when compared with *Usp11*^*+/+*^ counterparts. *B*, quantification of E-cadherin strongly positive cells in mammary glands in (*A*). The results represent the mean ± SEM of seven individual mice. ∗*p* < 0.05 vs the *Usp11*^*+/+*^ group. *C*, Western blotting analysis of the mammary glands from 5-week-old *Usp11*^*+/+*^ and *Usp11*^*−/−*^ mice. β-Actin was used as an internal control. *D*, quantification of the expression of E-cadherin and Vimentin in (*C*). The results represent the mean ± SEM of four individual samples. ∗*p* < 0.05 vs the *Usp11*^*+/+*^ group. *E*, representative western blotting analysis of the primary MECs isolated from *Usp11*^*+/+*^ and *Usp11*^*−/−*^ mice. β-Actin was used as an internal control. *F*, quantification of the expression of E-cadherin and Vimentin in (*E*). The results represent the mean ± SEM of four individual samples. ∗*p* < 0.05 vs the *Usp11*^*+/+*^ group. *G*, representative western blotting analysis of murine luminal tumor cells infected with sh-Ctrl, sh-Usp11#1, or sh-Usp11#2 lentivirus. GAPDH was used as an internal control. *H*, representative western blotting analysis of the murine luminal mammary tumor cells infected with Flag, Flag-USP11, or Flag-USP11-C318A lentivirus. GAPDH was used as an internal control.

We and others previously reported that the deletion of some DNA damage repair genes not only induces defective DNA damage repair but also causes aberrant differentiation in mammary epithelial and cancerous cells (6, 7, 13, 14, 15, 19). We further examined this in Usp11-deficient MECs. We examined the expression of E-cadherin, a profound marker for luminal and epithelial cells (36), and Vimentin, a mesenchymal marker that is not expressed in luminal MECs (37). We found that the number of E-cadherin strongly positive MECs in *Usp11*^*−/−*^ mice was significantly less than in *Usp11*^*+/+*^ mice (Figs. 2, *A*, *B* and S2*A*). The intensity of E-cadherin staining in *Usp11*^*−/−*^ MECs was also drastically reduced relative to that in *Usp11*^*+/+*^ counterparts (Figs. 2*A* and S2*A*). No clear increase of Vimentin was detected in *Usp11*^*−/−*^ MECs when compared with *Usp11*^*+/+*^ MECs (Fig. 2*A*). In accordance with the findings derived from IHC analysis, western blotting analysis also revealed that the expression of E-cadherin, but not Vimentin, was significantly reduced in *Usp11*^*−/−*^ mammary tissues when compared with *Usp11*^*+/+*^ tissues (Fig. 2, *C* and *D*). Taking advantage of the low-serum cell culture system that maintains the luminal cell fate of primary mammary epithelial and cancerous cells *in vitro* (7), we isolated and cultured primary mouse MECs from 5-week-old mice. We found that, relative to *Usp11*^*+/+*^ MECs, *Usp11*^*−/−*^ MECs again expressed a significantly reduced level of E-cadherin, but not Vimentin (Fig. 2, *E* and *F*), confirming that the loss of Usp11 inhibits the expression of E-cadherin in MECs. Notably, *Usp11*^*−/−*^ MECs expressed comparable Cdh1 mRNA levels relative to *Usp11*^*+/+*^ MECs (Fig. S2*B*), suggesting that the reduction of E-cadherin protein level by Usp11 loss in MECs is unlikely regulated at the transcriptional level.

We then performed whole-mount analysis for mouse mammary glands at different ages. We found that *Usp11*^*−/−*^ female mice developed normally as WT did in the puberty stage (Fig. S1, *B* and *C*). Furthermore, *Usp11*^*−/−*^ mammary glands also developed normally during pregnancy and had no problem in feeding pups (data not shown). These data suggest that Usp11 is dispensable for mammary gland development and that Usp11 loss reduced E-cadherin is not sufficient to result in developmental defects. Taken together, these results indicate that loss of Usp11 inhibits the expression of E-cadherin in MECs.

Several groups indicate that P-cadherin or N-cadherin, both of which belong to the cadherin protein family, are sometimes up-regulated, providing compensatory functions for the loss of E-cadherin (38, 39, 40, 41). Our data showed that the expressions of P-cadherin and N-cadherin were both drastically reduced in *Usp11*^*−/−*^ mammary glands (Fig. S2*D*). Interestingly, the expressions of β-catenin and α-catenin, which combine with the intracellular domain of E-cadherin to form a complex, were also reduced in *Usp11*^*−/−*^ mammary glands when compared to *Usp11*^*+/+*^ glands (Fig. S2*D*). These data indicate that USP11 also positively regulates the expression of P-cadherin and N-cadherin, as well as β-catenin and α-catenin, suggesting that the reduction of E-cadherin by Usp11 loss in mammary glands does not lead to compensatory increase of P-cadherin and N-cadherin.

Loss of luminal features of tumor cells is one of the major causes promoting mammary tumor cell dedifferentiation and stimulating tumor-initiating and metastatic potential. We discovered that loss of Usp11 reduced the expression of E-cadherin in MECs; therefore, we wanted to investigate if Usp11 controls the differentiation of luminal-type breast cancer cells. To test this hypothesis, we took advantage of the MMTV-PyMT luminal-type mammary tumor cell and transplantation model system we established (7, 19). We knocked down (KD) Usp11 in luminal tumor cells using lentiviruses. We found that, relative to control, Usp11 KD clearly reduced the expression of E-cadherin in luminal tumor cells (Figs. 2*G* and S2*C*). This inhibitory effect on E-cadherin expression by Usp11 KD was also observed in mouse HC11 cells (Fig. S2*E*). We then ectopically expressed WT USP11 (Flag-USP11) and an enzymatically inactive mutant form of USP11, USP11-C318 A (Flag-USP11-C318 A), in luminal tumor cells. Western blotting analysis revealed that, relative to control (Flag), overexpression of Flag-USP11, but not Flag-USP11-C318 A, significantly enhanced the expression of E-cadherin in tumor cells (Figs. 2*H* and S2*F*). Notably, over-expression of either Flag-USP11 or Flag-USP11-C318 A did not induce significant changes in Cdh1 mRNA expression (Fig. S2*G*). Together, these results demonstrated that Usp11 promotes the protein expression of E-cadherin dependent on its deubiquitinase activity.

Loss of E-cadherin accompanying weakened adhesion phenotype in tumor cells is one of the major features when a carcinoma deteriorates. As USP11 positively regulated the E-cadherin protein in mammary tumor cells, it was of interest to know whether USP11 regulated mammary tumor cell adhesion. We transfected Flag-E-cadherin and Flag into sh-Usp11 and sh-control cells, respectively. We found that the adhesion ability of Flag-overexpressing sh-Usp11 cells was significantly less than that of Flag-overexpressing sh-control cells. Notably, the adhesion ability of E-cadherin-overexpressing sh-Usp11 cells was significantly higher than that of Flag-overexpressing sh-Usp11 but significantly less than that of E-cadherin-overexpressing sh-control cells (Fig. S2, *H* and *I*). These results indicate that deficiency of USP11 in tumor cells reduces the adhesion ability, which is partially restored by ectopic expression of E-cadherin. These data suggest that E-cadherin plays a critical role in USP11-mediated breast cancer cell adhesion.

Interestingly, we observed that knockdown of Usp11 reduced the expression of β-catenin or α-catenin. The level of β-catenin or α-catenin in Flag-E-cadherin expressing sh-Ctrl cells is comparable to that in Flag-expressing sh-Ctrl cells, and in Flag-E-cadherin expressing sh-Usp11 cells is also comparable to that in Flag-expressing sh-Usp11 cells (Fig. S2*H*). These results show that overexpression of E-cadherin in mammary tumor cells does not induce the expression of β-catenin or α-catenin, nor rescues the reduction of β-catenin or α-catenin by Usp11 deficiency, which suggests that Usp11 deficiency reduces the expression of β-catenin or α-catenin independent of the decrease of E-cadherin.

### USP11 binds to E-cadherin

**Figure 3:**
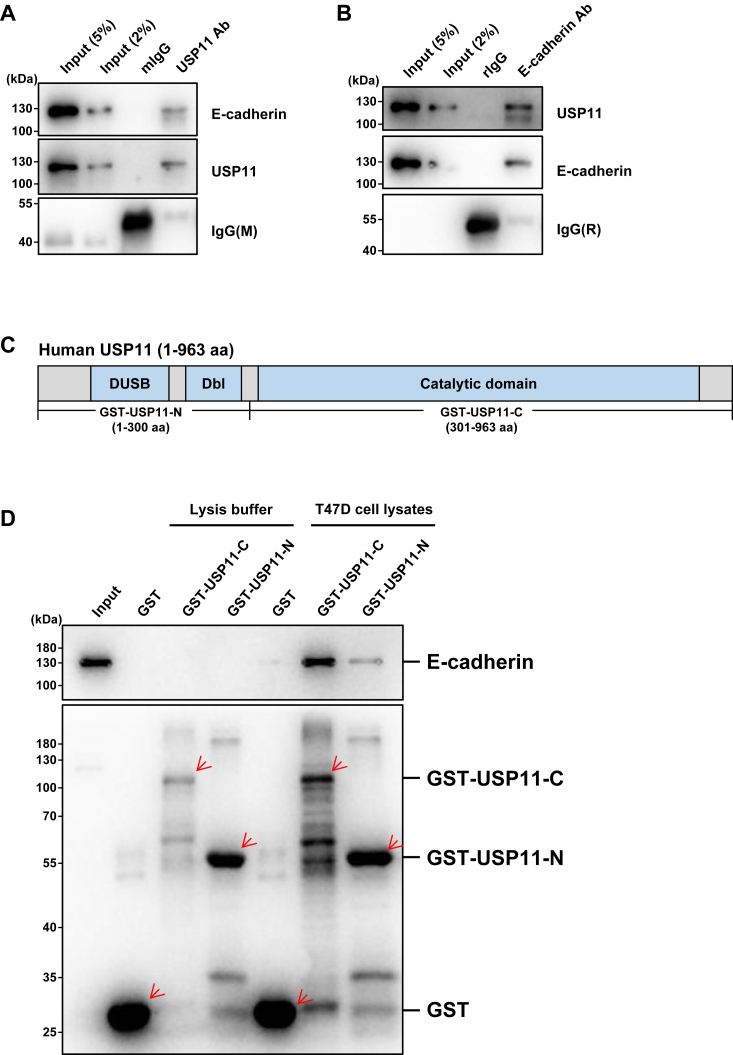
**USP11 interacts with E-cadherin *in vivo* and *in vitro*.***A*, T47D cell lysates were immunoprecipitated with an antibody against USP11 or control IgG and analyzed by western blotting. *B*, T47D cell lysates were immunoprecipitated with an antibody against E-cadherin or control IgG and analyzed by western blotting. *C*, Schematic representation of the USP11 fragments that were used in the GST pull-down assay. DUSP: domain present in ubiquitin-specific proteases; UBL: ubiquitin-like domain. *D*, purified GST or GST-tagged N-terminal and C-terminal fragments of USP11 were used to interact with E-cadherin in T47D cell lysates, and the eluted proteins were analyzed by western blotting using GST or E-cadherin primary antibodies.

Prompted by the findings that Usp11 regulates the expression of E-cadherin in mammary epithelial and cancerous cells and that USP11 functions as a deubiquitinating enzyme, we hypothesize that USP11 might be the deubiquitinase of E-cadherin. To test this hypothesis, we checked the interaction between USP11 and E-cadherin in a human luminal-type breast cancer cell line, T47D. We found that endogenous E-cadherin was co-immunoprecipitated with USP11 (Fig. 3*A*), and reciprocally, endogenous USP11 was also co-immunoprecipitated with E-cadherin (Fig. 3*B*), indicating that USP11 and E-cadherin bind to each other in human breast cancer cells. The *in vivo* interaction between Usp11 and E-cadherin was also observed in mouse mammary tumor cells (Fig. S3*A*). In addition, we detected the interaction between USP11 and E-cadherin by immunoprecipitation assay using Flag antibody in Flag-USP11 overexpressed HEK293 T cells or T47D cells (Fig. S3, *B* and *C*).

Since E-cadherin is a transmembrane protein holding a cytoplasmic domain for signal transduction and interacting with intracellular proteins, such as p120 and β-catenin (42, 43), we wondered whether USP11 bound to the E-cadherin cytoplasmic region. We then performed the co-immunoprecipitation assay and confirmed that USP11 could bind to E-cadherin-C (731-882aa), the cytoplasmic region of E-cadherin (Fig. S3*D*).

To further confirm the interaction between USP11 and E-cadherin, we generated GST fusion proteins containing N-terminal (residues 1–300 amino acids, termed GST-USP11-N) and C-terminal (residues 301–963 amino acids, termed GST-USP11-C) sequences of USP11 (Fig. 3*C*). GST pull-down assay was carried out using the GST, GST-USP11-N, or GST-USP11-C purified proteins in T47D cell lysates or lysis buffer, respectively. We found that GST-USP11-C readily interacted with endogenous E-cadherin, whereas GST-USP11-N weakly and GST did not bind to E-cadherin (Figs. 3*D* and S3*E*). Together, these results indicate that USP11 binds to E-cadherin and that this interaction is mainly mediated by the C-terminal region of USP11.

### USP11 deubiquitinates E-cadherin at K738

**Figure 4:**
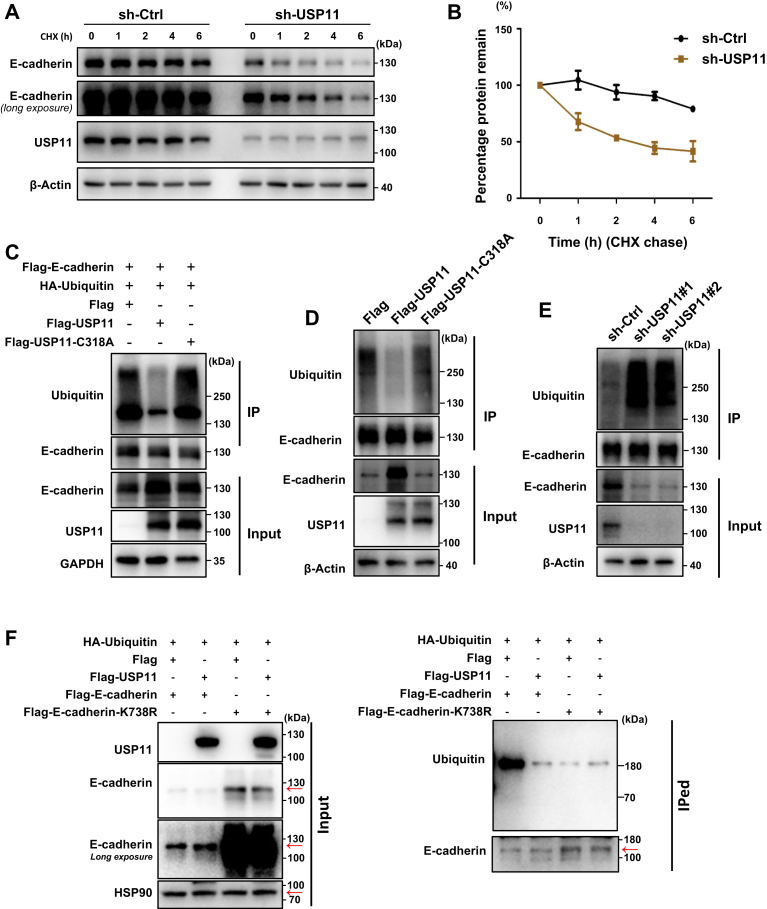
**USP11 is a deubiquitinase for E-cadherin**. *A*, sh-Ctrl or sh-USP11 infected T47D cells were treated with 3 μM CHX for 0, 1, 2, 4, or 6 h. The protein expression of E-cadherin and USP11 was then detected by Western blotting. β-Actin was used as an internal control. *B*, the relative protein levels of E-cadherin from four independent experiments of (*A*) were statistically analyzed. *C* and *F*, HEK293T cells were transfected with Flag-E-cadherin, Flag-E-cadherin-K738R, HA-Ubiquitin, Flag-USP11, Flag-USP11-C318A, or Flag for 24 h, treated with MG132 (20 nM) for additional 6 h, and then lysed. Immunoprecipitation was performed using E-cadherin rabbit polyclonal antibody in the cell lysates. Ubiquitin and E-cadherin in immunoprecipitates and E-cadherin and USP11 in input samples were detected by Western blotting. GAPDH (*C*) or HSP90 (*F*) was used as an internal control for input samples. *D* and *E*, T47D cells infected with Flag, Flag-USP11, or Flag-USP11-C318A lentivirus for 48 h (*D*) or infected with sh-USP11#1, sh-USP11#2, or sh-Ctrl lentivirus for 48 h (*E*) were treated with MG132 (20 nM) for 6 h and then lysed. Immunoprecipitation was taken using E-cadherin rabbit polyclonal antibody in the cell lysates. Ubiquitin and E-cadherin in immunoprecipitates, and ubiquitin, E-cadherin, and USP11 in input samples were detected by Western blotting.

To examine whether USP11 deubiquitinates E-cadherin, we checked the effect of USP11 deficiency on E-cadherin protein turnover. Cycloheximide (CHX) chase experiments showed that the depletion of USP11 accelerated the degradation of E-cadherin protein in T47D cells compared to the control group (Figs 4, *A* and *B*). Then, we performed a ubiquitin assay for E-cadherin. We transfected HEK293 T cells with Flag, Flag-USP11, or Flag-USP11-C318 A along with Flag-E-cadherin and HA-ubiquitin, and then immunoprecipitations were performed using an E-cadherin antibody in these cell lysates. We found that overexpression of Flag-USP11 drastically reduced the level of the ubiquitinated form of E-cadherin compared to that of the Flag control; however, overexpression of Flag-USP11-C318 A did not change the level of the ubiquitinated form of E-cadherin relative to that of the Flag control (Figs 4*C* and S4*A*). Next, we checked the effect of USP11 on the ubiquitination of endogenous E-cadherin. We observed that overexpression of USP11, but not the enzyme inactive form, C318 A mutant, reduced the level of the ubiquitinated form of E-cadherin in T47D cells compared to Flag control (Fig. 4*D*). Additionally, we observed that depletion of USP11 enhanced the level of the ubiquitinated form of E-cadherin in human T47D cells (Fig. 4*E*) or mouse luminal cancerous cells (Fig. S4*B*) compared to the control.

Hartsock and Nelson have discovered that K754 and K833 in the JuxtaMembrane Domain (JMD) of mouse E-cadherin are required for ubiquitination and proteasomal degradation of JMD (44). K754, but not K833, is required for ubiquitination and degradation of full-length E-cadherin (44). We, therefore, determined the role of K738 in human E-cadherin, equivalent to K754 in mouse E-cadherin (Fig. S4*C*), in regulating the ubiquitination of E-cadherin. We transfected equal amounts of Flag-E-cadherin and Flag-E-cadherin-K738 R cDNA into 293T cells and found that Flag-E-cadherin-K738 R protein level was higher than Flag-E-cadherin level, suggesting that E-cadherin-K738 R protein is more stable than E-cadherin. Importantly, the level of ubiquitinated E-cadherin-K738 R was drastically reduced compared to that of ubiquitinated E-cadherin-WT. The addition of USP11 reduced the level of ubiquitinated E-cadherin-WT but not that of ubiquitinated E-cadherin-K738 R (Fig. 4*F*). These results not only confirm that K738 is required for ubiquitination of full-length human E-cadherin but also suggest that USP11 deubiquitinates E-cadherin at K738 (removes ubiquitin from E-cadherin on K738).

As USP11 binds to and regulates P-cadherin and N-cadherin, and JMD including the K738 ubiquitin sites of E-cadherin was conserved in P-cadherin and N-cadherin (Fig. S3*F*), we also performed the ubiquitin assay and discovered that overexpression of USP11 in T47D cells reduced the levels of ubiquitinated P-cadherin and N-cadherin (Fig. S3*G*), suggesting that USP11 also deubiquitinates P-cadherin and N-cadherin.

### Overexpression of WT USP11, but not the USP11-C318 A mutant, promotes luminal differentiation and DNA damage repair, suppressing tumorigenesis

**Figure 5:**
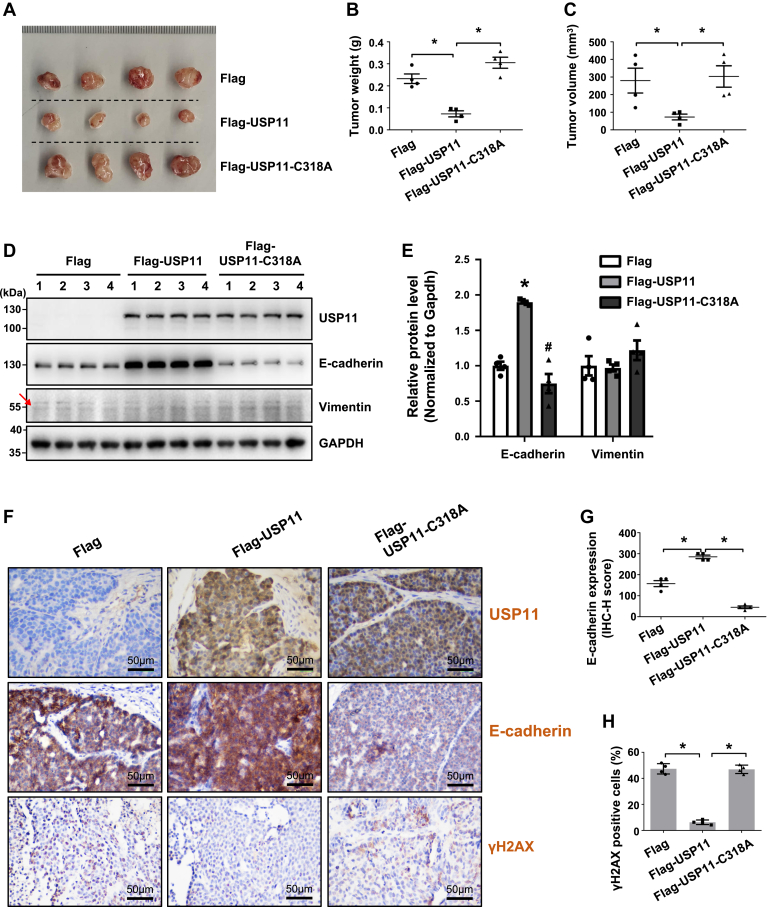
**WT USP11, not the USP11-C318A mutant, inhibits tumor cell proliferation and promotes luminal differentiation in suppression of tumorigenesis.** 5 × 10^5^ murine luminal tumor cells infected with Flag-USP11, Flag-USP11-C318A, or Flag lentivirus were transplanted into the inguinal mammary fat pad of NSG mice (n = 4 for each group). After 20 days, mice were sacrificed to obtain the regenerated tumors. *A–C*, gross appearance (*A*), weight (*B*), and volume (*C*) of the xenograft tumors were determined. Data in (*B*) and (*C*) are presented as the mean ± SEM of four samples. ∗*p* < 0.05. *D*, Western blotting analysis of the tumor samples in (A). GAPDH was used as an internal control. *E*, quantification of the expression of E-cadherin and Vimentin in tumor samples in (*D*). The results represent the mean ± SEM of four individual samples. ∗*p* < 0.05 vs Flag group; ^#^*p* < 0.05 vs Flag-USP11 group. *F*, representative IHC staining of tumor samples in (*A*). *G* and *H*, quantification of expression levels of E-cadherin (*G*) and γH2AX-positive cells (*H*) in tumors in (*F*). The results represent the mean ± SEM of four individual samples. ∗*p* < 0.05.

Inspired by the findings that Usp11 controls the luminal features of mammary cells, we next examined whether Usp11 functions similarly *in vivo* in tumorigenesis using a xenograft mouse model. We found that both the weight and volume of Flag-USP11-expressing tumors were significantly less than those of Flag-expressing tumors (Fig. 5, *A*–*C*). Cohorts from the Flag-USP11-C318A-expressing tumors exhibited weights and sizes that were not significantly different compared to those of Flag-expressing tumors (*p* > 0.05) but were significantly larger than those of Flag-USP11-expressing tumors (Fig. 5, *A*–*C*). These data indicate that WT USP11, but not the USP11-C318 A mutant, suppresses tumorigenesis. Next, we analyzed the protein levels in these tumors by western blotting, and we found that, compared to control (Flag) tumors, Flag-USP11-expressing tumors, but not Flag-USP11-C318A-expressing tumors, displayed significantly increased levels of E-cadherin (Fig. 5, *D* and *E*). Interestingly, we failed to detect a clear difference in the expression of Vimentin among these tumors (Fig. 5, *D* and *E*). We then performed IHC analysis and found that, in comparison with the cells in control (Flag) tumors, cells in Flag-USP11-expressing tumors, but not the cells in Flag-USP11-C318A-expressing tumors, exhibited significantly enhanced levels of E-cadherin (Figs. 5, *F* and *G* and S5*A*). In addition, we detected that γH2AX-positive cells in Flag-USP11-tumors were significantly fewer than those in Flag-tumors or Flag-USP11-C318A-tumors (Figs. 5, *F* and *H* and S5*B*), confirming the role of WT USP11, not the USP11-C318 A mutant, in promoting DNA damage repair in tumorigenesis. In sum, these results indicate that Usp11 prevents E-cadherin from degradation and promotes DNA damage repair to suppress tumorigenesis, which is dependent on its deubiquitinase activity.

### Expression of USP11 is positively correlated with that of E-cadherin in human breast cancer and is predictive of patient outcome

**Figure 6:**
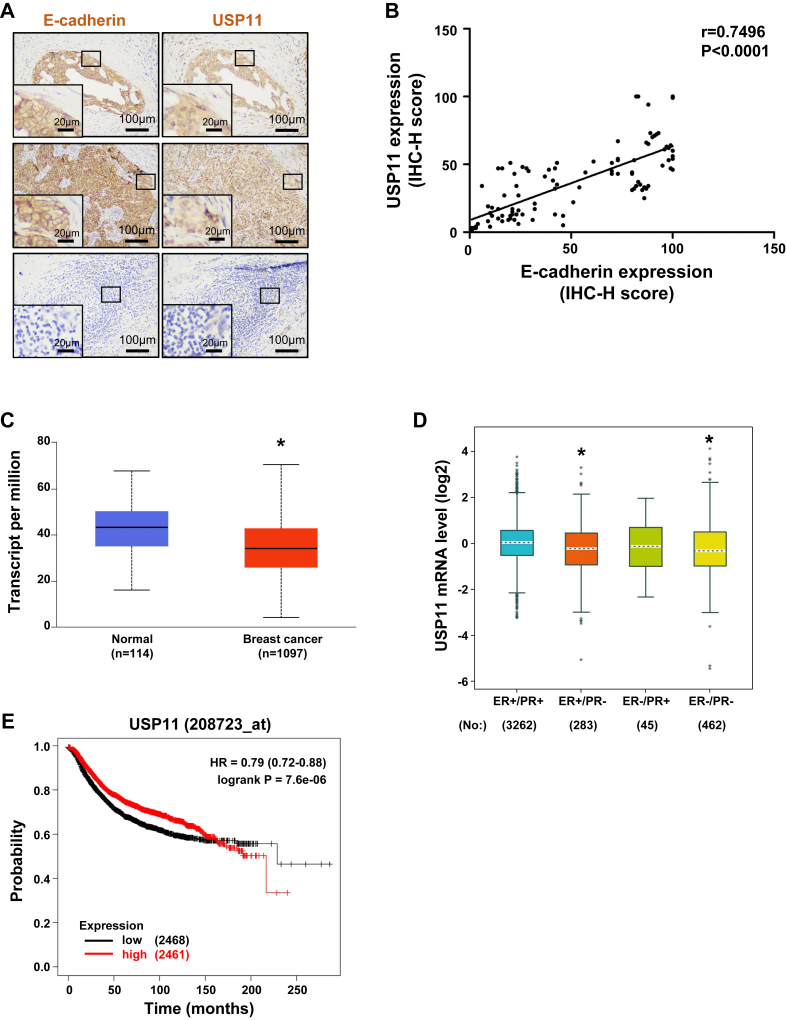
**Correlation analysis of USP11 with E-cadherin in human breast cancers.***A*, representative IHC analysis of E-cadherin and USP11 in human breast cancer samples. *B*, correlation analysis of E-cadherin and USP11 protein levels for human breast cancer samples. n = 100. *C*, analysis of USP11 mRNA levels in human breast cancers in the Clinical Proteomic Tumor Analysis Consortium (CPTAC) breast cancer dataset (http://ualcan.path.uab.edu/analysis.html). Normal, normal breast; Breast cancer, breast invasive carcinoma. ∗*p* < 0.05 vs Normal group. *D*, analysis of USP11 mRNA expression in different types of human breast tumors according to the IHC staining of ER and PR (http://bcgenex.ico.unicancer.fr). ∗*p* < 0.05 vs ER+/PR + group. *E*, Kaplan-Meier recurrence-free survival curve of USP11 in breast cancer patients.

We screened 30 human invasive breast cancer samples and then selected 5 ER-positive and 5 ER-negative samples that were equal to or larger than 10 mm × 10 mm x 5 mm (length x width x height) in size. In addition, only samples with a consistent tumor cell content >60% of tissues were selected and analyzed. We performed HE staining and IHC with an antibody against E-cadherin as the first round of analysis. We found that the tumors displayed drastic inter- and intra-tumoral heterogeneity. Most, if not all, tumors consisted of multiple different types of sub-tumor nodules/foci, as evidenced by HE-based pathological morphology and IHC-based E-cadherin positivity, which is not significantly associated with clinically diagnosed ER status (Figs. 6*A* and S6, *A*–*J*). Due to the highly heterogeneous positivity of E-cadherin in multiple sub-tumor nodules/foci of each individual tumor, each sub-nodule/focus that was larger than 500 μm and had no boundary link with another sub-nodule/focus was analyzed and regarded as a separate tumor nodule/focus. Because of the inability of immunofluorescent double staining of USP11 and E-cadherin antibodies in tumor samples, we performed IHC analysis with antibodies against USP11 and E-cadherin for serial sections of the samples. We carefully analyzed the positivity of USP11 and E-cadherin by H scores as well as their correlation in every tumor nodule/focus. We found that USP11 was readily detected in E-cadherin-positive tumor cells but hardly detectable or weakly expressed in E-cadherin-negative tumor cells (Figs. 6*A* and S6, *A*–*J*). Of 100 tumor nodules/foci analyzed, a significant positive relationship between USP11 and E-cadherin H-scores was detected (Fig. 6*B*). Together, these clinical findings are consistent with our results in mice, suggesting an opportunity to use murine systems to further explore how USP11 regulates E-cadherin to control human breast biology as well as cancer development and progression.

Next, we checked the mRNA expression of USP11 in the breast cancer patient sample sets. We found a significant reduction in USP11 mRNA expression in primary tumors compared to that in normal breasts (Fig. 6*C*). In human breast tumor samples, the mRNA expression of USP11 in ER- and PR-double-negative (ER-/PR-) tumors was significantly lower than that in ER- and PR-double-positive (ER+/PR+) tumors (Fig. 6*D*), showing a decreased expression of USP11 in advanced breast tumors. Kaplan-Meier analysis of recurrence-free survival revealed that the expression of USP11 genes was significantly predictive of patient outcome. High USP11 expression predicted better patient outcomes (Fig. 6*E*). Consistent with our results in mice, these data indicate that the expression of USP11 is positively correlated with E-cadherin in human breast cancers and that USP11 functions as a tumor suppressor in breast cancer.

## Discussion

In this article, we found that the deletion of Usp11 in mice reduced the expression of E-cadherin and promoted DNA damage in MECs. Overexpression of WT USP11, not a deubiquitinase-inactive mutant form of USP11, promoted luminal differentiation, enhanced DNA damage repair, and suppressed tumorigenesis. Mechanistically, we found that Usp11 stimulated the expression of E-cadherin dependent on its deubiquitinase activity and that USP11 deubiquitinated E-cadherin at K738. We discovered that USP11 is bound to E-cadherin through its C-terminal region. In human breast cancers, the expression of USP11 was positively correlated with that of E-cadherin and high USP11 predicted better recurrence-free survival. Our findings provide compelling genetic and biochemical evidence that USP11 not only promotes DNA damage repair but also deubiquitinates E-cadherin and maintains the luminal features of mammary tumor cells, leading to the suppression of breast cancer.

The function of USP11 in controlling tumor development and progression is very puzzling. USP11 may stimulate tumorigenesis by deubiquitinating cIAP2, NONO, NF90, E2F1, or cytoplasmic p21; however, it may also inhibit tumorigenesis through stabilizing PML, Mgl-1, PTEN, ARID1A, or nuclear p21 (29, 30). The findings that USP11 stimulates tumorigenesis have been exclusively obtained from artificial overexpression or knockdown of USP11 in various cancer cell lines, in which multiple oncogenic proteins have been identified as its substrates. It is not surprising that USP11, a deubiquitinase widely expressed in multiple tissues or cell lineages, has many substrates including oncogenic proteins; however, it remains to be investigated whether, under physiological conditions, USP11 promotes tumorigenesis through deubiquitinating those substrates *in vivo*. Although most results on the tumor suppressive role of USP11 were also derived from *in vitro* cell culture systems, a finding that the loss of one allele of Usp11 in male mice destabilizes PTEN to promote prostate epithelial cell tumorigenesis provides genetic evidence that USP11 suppresses prostate tumorigenesis in males (29). Notably, USP11 is an X chromosome-linked gene; it is, therefore, expected that the loss of both alleles of Usp11 in females regulates tumorigenesis through distinct mechanisms. Interestingly, USP11 may also inhibit breast cancer cell proliferation through deubiquitinating PTEN (31); however, no genetic evidence indicates that USP11 suppresses mammary tumorigenesis. In the present study, we found that overexpression of WT USP11, not mutant USP11, suppressed luminal breast cancer cell tumorigenesis. Our findings demonstrate that USP11 is a tumor suppressor for luminal breast cancer.

E-cadherin is predominantly expressed in normal luminal epithelial cells and luminal breast cancer cells. It has been well documented that E-cadherin is a tumor suppressor and a profound marker of mammary luminal epithelial and cancerous cells. Germline deletion or mutation in CDH1 is associated with breast cancer development, and deregulation of E-cadherin in breast cancer shows a worse prognosis and shortened overall survival (45). Consistently, a high level of E-cadherin predicts better survival for breast cancer patients and overexpression of E-cadherin in mice or cancer cells suppresses breast tumorigenesis (17, 46). Obviously, maintaining the expression or preventing the loss of E-cadherin in breast cancer cells is expected to bring outstanding outcomes in breast cancer treatment (47). CDH1/E-cadherin can be regulated at both transcriptional and post-transcriptional levels. GATA3 promotes and most EMT-TFs, including SNAIL, SLUG, and TWIST, suppress the transcription of CDH1 (19, 20, 21, 22, 23), while Hakai and MDM2 function as ubiquitin E3 ligases promoting degradation of E-cadherin (24, 25). It is unknown how E-cadherin is deubiquitinated. We found that Usp11 was predominantly expressed in luminal epithelial cells in mice, and the protein levels of Usp11 and E-cadherin were positively correlated in human breast cancer samples. We observed that the loss or depletion of Usp11 eliminated E-cadherin expression in MECs and tumor cells, and overexpression of WT USP11, not a deubiquitinase-inactive mutant form of USP11, promoted the expression of E-cadherin protein in tumor cells *in vitro* and *in vivo*. We revealed that USP11 bound to and deubiquitinated E-cadherin. Our findings demonstrated that USP11 stabilizes E-cadherin and maintains or promotes the luminal features of mammary epithelial and cancerous cells. Our results identified USP11 as the first deubiquitinase of E-cadherin, indicating that the stability of E-cadherin protein is collaboratively controlled by E3 ubiquitin ligases and USP11 deubiquitinase.

Deficiency of BRCA1 and a few BRCA1 interacting proteins including FANCD2, BRG1, NUMB, and HES1, induces defective DNA damage repair, loss of luminal differentiation, and aberrant luminal-to-basal differentiation in immortalized MECs (14, 15). Interestingly, depletion of another BRCA1-interacting protein, BRCA2, results in defects of DNA repair but not that of differentiation in mammary cells (14), which suggests that not all intrinsic DNA repair defects induced by deficiency of BRCA1-interacting proteins promote or are associated with aberrant differentiation.

In this study, we discovered that loss of USP11 reduced the expression of E-cadherin and induced DNA damage in MECs and that overexpression of USP11 promoted luminal differentiation, enhanced DNA damage repair, and suppressed tumorigenesis. Our findings support that USP11, a BRCA1-interacting protein, not only repairs DNA damage but also controls the differentiation of mammary tumor cells. Whether other BRCA1-interacting proteins or DNA damage repair proteins function similarly in controlling mammary tumor cells remains to be investigated.

## Experimental procedures

### Generating Usp11 knock-out mice

The targeting construct was generated to delete a genomic fragment containing exons two to four of the mouse Usp11 gene by Shanghai Model Organisms Center. Briefly, a mouse genomic DNA fragment spanning the Usp11 locus was isolated by PCR from mouse embryonic stem (ES) cell genomic DNA. LoxP sites were introduced flanking exons two and four of Usp11. A phosphoglycerate kinase (PGK) promoter-driven neomycin (neo) cassette flanked by frt sites was inserted upstream of the loxP site upstream of exon 5, and a PGK promoter-driven thymidine kinase (Tk) gene was inserted downstream of exon 12. The targeting construct was electroporated into ES cells and selected with G418 and ganglocyclovir. Doubly resistant clones were screened for homologous recombination events. *Usp11*^*flox/+*^ ES clones were injected into BL/6 blastocysts, and chimeric mice were crossed with BL/6 mice to generate *Usp11*^*flox/+*^ heterozygotes. Germline-transmitted *Usp11*^*flox/+*^ mice were then crossed with EIIa-Cre transgenic mice [B6.FVB-Tg (EIIa-Cre); Jackson Laboratory] to generate *Usp11*^*+/−*^ heterozygotes, which were then intercrossed to produce *Usp11*^*−/−*^ mice. Successful deletion of exons two to four and the protein expression in *Usp11*^*−/−*^ mice was confirmed by PCR and Western blotting (Fig. 1, *C* and *D*). Mice were housed in a 12/12 light cycle, pathogen-free environment and allowed to get water and food freely. All animal experiments were approved by the Institutional Animal Care and Use Committee at Shenzhen University.

### Histopathology, immunostaining, and mammary gland whole-mount staining

Histopathology and immunostaining were performed by standard procedures as previously described (6, 13). The primary antibodies used for immunostaining were USP11 (1:100 dilution, sc-365528, Santa Cruz), E-cadherin (1:1000 dilution, ab109232, Abcam), γH2AX (1:2000 dilution, ab26350, Abcam), and Vimentin (1:1000 dilution, 5741S, CST). The tissue sections stained with primary antibodies were incubated with MaxVision HRP-Polymer anti-Mouse/Rabbit IHC Kit (Maixin Biotech) and visualized by DAB kit (Maixin Biotech) according to the manufacturer’s instructions. Cell nuclei were visualized using hematoxylin. The expression of E-cadherin in tumors from immunohistochemistry (IHC) staining was quantified by IHC-H score, as described previously (48). The mammary glands from 5-week-old *Usp11*^*+/+*^ and *Usp11*^*−/−*^ female mice were visualized by carmine staining as described previously (49).

### Cell culture

The primary mouse MECs were prepared from 5-week-old *USP11*^*+/+*^ and *Usp11*^*−/−*^ female mice as previously described (6). The primary mouse mammary luminal tumor cells were isolated from mammary tumors developed in 6-week-old *MMTV-PyMT* female mice. Both cell lines were maintained in low serum culture medium (DMEM/F12 medium (Gibco) with 10 ng/ml of EGF (Gibco), 10 μg/ml of insulin (Gibco), 1% bovine serum albumin (Gibco), 2% calf serum (Gibco), 100 U/ml penicillin, and 100 μg/ml streptomycin (Gibco) (7). T47D and HEK293 T cells, which were purchased from the American Type Culture Collection (ATCC), were tested, authenticated, and maintained under the ATCC recommendations (6).

### Gene knockdown and overexpression

For gene knock-down, four 21 bp long oligos targeting different sequences of the human USP11 or mouse Usp11 gene and one control oligo were constructed into third-generation pLKO.1-puro vector (Addgene) as pLKO.1-puro-sh-USP11#1 (sh-USP11#1), pLKO.1-puro-sh-USP11#2 (sh-USP11#2), pLKO.1-puro-sh-Usp11#1 (sh-Usp11#1), pLKO.1-puro-sh-Usp11#2 (sh-Usp11#2), and pLKO.1-puro-sh-control (sh-Ctrl), separately. These shRNA sequences were obtained from Hairpins RNAi Design Tools (https://www.broadinstitute.org/) and listed as follows: Human USP11#1: 5′-TCGCGGTTTCCAACCATTATG-3’; Human USP11#2: 5′-CCCTCCCTTCTAGTCTTTATT-3’; Mouse Usp11#1: 5′-CCTACTATGGTCTGATACTTT-3’; Mouse Usp11#2: 5′-GCAGCCTATGTCTTGTTCTAT-3’. The control oligo sequence was 5′-TTCTCCGAACGTGTCACGTTT-3’. cDNAs encoding E-cadherin, E-cadherin-K738 R, E-cadherin-C (731-882aa), USP11, and USP11-C318 A were cloned into pLVX-IRES-Puro-3xFlag (Addgene) vector as pLVX-IRES-Puro-Flag-E-cadherin (Flag-E-cadherin), pLVX-IRES-Puro-Flag-E-cadherin-K738 R [Flag- E-cadherin-K738 R], pLVX-IRES-Puro-Flag-E-cadherin-C (731-882aa) (Flag-E-cadherin-C), pLVX-IRES-Puro-Flag-USP11 (Flag-USP11), and pLVX-IRES-Puro-Flag-USP11-C318 A (Flag-USP11-C318 A), respectively. The pLKO.1-puro or pLVX-IRES-Puro-3xFlag vector was used as a backbone plasmid and was co-transfected with packaging plasmid - psPAX2 (Addgene) and envelope plasmid - pMD2.G (Addgene), in a ratio of 9:6:3, into HEK293 T cells to package lentivirus. After 2 days of lentivirus infection, mammary tumor cells were selected with 1000 ng/ml puromycin for 5 days and then maintained in a culture medium with 50 ng/ml puromycin for further use.

### Transplantation model of mammary tumors

For the transplantation of mouse mammary tumor cells, 5 × 10^5^ cells infected with Flag, Flag-USP11, or Flag-USP11-C318 A lentivirus were injected into the left or right inguinal mammary fat pads of 4-week-old female immunodeficient NCG mice. After 20 days of transplantation, mice were sacrificed and mammary tumors were dissected for analysis.

### Western blotting, Co-immunoprecipitation (Co-IP), GST pull-down, and qRT-PCR

For western blotting, proteins from cultured cells, mammary gland tissues, or tumors were prepared using SDS Lysis Buffer (P0013 G, Beyotime) according to the manufacturer’s instructions. 20 μg of protein from each sample was loaded for analysis. The primary antibodies for western blotting were listed as follows: USP11 (1:500 dilution, Santa Cruz), E-cadherin (1:2000 dilution, CST), Vimentin (1:2000 dilution, CST), GST (1:2000 dilution, AF2299, Beyotime, China), GAPDH (1:2000 dilution, AF0006, Beyotime), and β-Actin (1:2000 dilution, AA128, Beyotime). Protein bands were visualized by BeyoECL SuperMoon Kit (P0018HS, Beyotime) using Tanon-5200Multi gel imaging system (Tanon) and quantified by Quantity one software (Bio-Rad). For Co-IP, 2 mg cell lysates prepared from IP cell lysis buffer (P0013, Beyotime) were incubated with anti-USP11 antibody (Santa Cruz), anti-Flag antibody (Sigma), anti-E-cadherin antibody (CST), mouse normal IgG (Santa Cruz), or rabbit normal IgG (CST) at 4 °C overnight. Then, the mixtures were incubated with Protein A  + G Agarose (P2055, Beyotime) for 1 h. After PBS washing, the agarose was boiled in 1×SDS loading buffer, and the USP11 and E-cadherin proteins were immunoblotted in these suspensions. For GST pull-down, cDNA sequence encoding N-terminus of USP11 (1–300 amino acid) or C-terminus of USP11 (301–963 amino acid) was constructed into pGEX-4T-1 (Addgene) vector. The GST and GST tag fusion proteins were induced in BL21(DE3) *Escherichia coli* (D0337, Beyotime) by 1 mM IPTG (ST098, Beyotime) at 16 °C in a shaker for 12 h. The E-cadherin protein for GST Pull-down assay was prepared from human luminal-type T47D cell lysates. The GST, GST-USP11-N, or GST-USP11-C fusion protein was incubated with T47D cell lysates or lysis buffer for 1 h, and the BeyoGold GST-tag Purification Resin (P2250, Beyotime) beads were added into the mixture for another 1 h of incubation. The beads were then washed with PBS twice, and the protein compounds integrated into the beads were immunoblotted with anti-GST or anti-E-cadherin antibody. qRT-PCR was conducted as previously reported (6). Briefly, total mRNA from cells was extracted using TaKaRa RNAiso Reagent (TaKaRa Biotechnology Co), and cDNA was synthesized from mRNA using T18 primer and the BeyoRT III First Strand cDNA Synthesis Kit (D7178S, Beyotime) according to the manufacturer’s instructions. Cdh1 mRNA expression level was detected by gene-specific primers.

### Ubiquitin assay

For detection of ubiquitination of exogenous E-cadherin, HEK293 T cells were transfected with pcDNA3-HA-ubiquitin (HA-ubiquitin, Dr Xingzhi Xu, Shenzhen University) (50), Flag-E-cadherin, Flag-E-cadherin-K738 R, Flag, Flag-USP11, or Flag-USP11-C318 A. The cells were then lysed, and the immunoprecipitations were performed using anti-E-cadherin antibody. The ubiquitin and E-cadherin levels in immunoprecipitated samples and the E-cadherin, USP11, and GAPDH levels in input samples were detected by immunoblotting. For detection of ubiquitination of endogenous E-cadherin, the lysates from human T47D cells infected with Flag, Flag-USP11, Flag-USP11-C318 A, sh-Ctrl, sh-USP11#1, or sh-USP11#2 lentiviruses, or from mouse mammary tumor cells infected with sh-Ctrl, sh-Usp11#1, or sh-Usp11#2 lentiviruses, were subjected to immunoprecipitation using anti-E-cadherin antibody. The ubiquitin and E-cadherin levels in immunoprecipitated samples and the Ubiquitin, E-cadherin, USP11, and β-Actin levels in input samples were detected by immunoblotting.

### Cell adhesion assay

For mammary tumor cell adhesion assay, a 12-well plate was coated with 10 ng/ml recombinant mouse E-cadherin protein (HY-P200008, MedChemExpress LLC) for 4 h. The cells were seeded into the plate at the amount of 100,000 cells per well, cultured for 12 h, and washed with PBS twice. The adhered cells in the plate were then fixed with paraformaldehyde, counted, and statistically analyzed.

### Human tumor samples and meta-analysis of gene expression data sets

De-identified Formalin-fixed paraffin-embedded (FFPE) human breast cancer samples were obtained from the Tissue Bank Core Facility at the University of Miami and Gansu Dian Medical Laboratory. Samples used for this study consisted of non-treated invasive breast carcinomas with known ER status, as previously reported (13, 51). Only samples with a consistent tumor cell content >60% of tissues were used for analysis. The Clinical Proteomic Tumor Analysis Consortium (CPTAC) breast cancer database was analyzed to compare the expression of USP11 mRNA in normal breasts and breast invasive carcinomas (http://ualcan.path.uab.edu/analysis.html). Breast Cancer Gene-Expression Miner v4.8 database (bcGenExMiner v4.8; http://bcgenex.ico.unicancer.fr) was analyzed to compare the expression of USP11 mRNA in ER-positive and ER-negative breast cancers. Prognostic values of USP11 expression were assessed by displaying the recurrence-free survival using the Kaplan-Meier plotter integrative data analysis tool (www.kmplot.com).

### Statistical analysis

Data were expressed as the mean ± SEM of the values for three or more independent experiments in each group. Two-tailed Student's *t* test was used for single or multiple comparisons in quantitative results. The Pearson correlation coefficient of USP11 and E-cadherin protein levels in human tumor samples was analyzed using GraphPad Prism software. *p* < 0.05 was considered statistically significant.

## Data availability

All the presenting data are available within the article or supplementary files.

## Supporting information

This article contains supporting information.

## Ethics approval

The Institutional Animal Care and Use Committee at Shenzhen University approved all animal procedures.

## Conflict of interest

The authors declare that they have no conflicts of interest with the contents of this article.

## References

[1] Hong R., Xu B. (2022). Breast cancer: an up-to-date review and future perspectives. Cancer Commun. (Lond).

[2] Kim M.J., Ro J.Y., Ahn S.H., Kim H.H., Kim S.B., Gong G. (2006). Clinicopathologic significance of the basal-like subtype of breast cancer: a comparison with hormone receptor and Her2/neu-overexpressing phenotypes. Hum. Pathol..

[3] Lim E., Vaillant F., Wu D., Forrest N.C., Pal B., Hart A.H. (2009). Aberrant luminal progenitors as the candidate target population for basal tumor development in BRCA1 mutation carriers. Nat. Med..

[4] Proia T.A., Keller P.J., Gupta P.B., Klebba I., Jones A.D., Sedic M. (2011). Genetic predisposition directs breast cancer phenotype by dictating progenitor cell fate. Cell Stem Cell.

[5] Molyneux G., Geyer F.C., Magnay F.A., McCarthy A., Kendrick H., Natrajan R. (2010). BRCA1 basal-like breast cancers originate from luminal epithelial progenitors and not from basal stem cells. Cell Stem Cell.

[6] Bai F., Smith M.D., Chan H.L., Pei X.H. (2013). Germline mutation of Brca1 alters the fate of mammary luminal cells and causes luminal-to-basal mammary tumor transformation. Oncogene.

[7] Bai F., Zheng C., Liu X., Chan H.L., Liu S., Ma J. (2022). Loss of function of GATA3 induces basal-like mammary tumors. Theranostics.

[8] Ignatiadis M., Sotiriou C. (2013). Luminal breast cancer: from biology to treatment. Nat. Rev. Clin. Oncol..

[9] Gao J.J., Swain S.M. (2018). Luminal A breast cancer and molecular assays: a review. Oncologist.

[10] Ades F., Zardavas D., Bozovic-Spasojevic I., Pugliano L., Fumagalli D., de Azambuja E. (2014). Luminal B breast cancer: molecular characterization, clinical management, and future perspectives. J. Clin. Oncol..

[11] Huang J., Li H., Ren G. (2015). Epithelial-mesenchymal transition and drug resistance in breast cancer (Review). Int. J. Oncol..

[12] Pooley K.A., Dunning A.M. (2019). DNA damage and hormone-related cancer: a repair pathway view. Hum. Mol. Genet..

[13] Bai F., Chan H.L., Scott A., Smith M.D., Fan C., Herschkowitz J.I. (2014). BRCA1 suppresses epithelial-to-mesenchymal transition and stem cell dedifferentiation during mammary and tumor development. Cancer Res..

[14] Wang H., Bierie B., Li A.G., Pathania S., Toomire K., Dimitrov S.D. (2016). BRCA1/FANCD2/BRG1-Driven DNA repair stabilizes the differentiation state of human mammary epithelial cells. Mol. Cell.

[15] Wang H., Xiang D., Liu B., He A., Randle H.J., Zhang K.X. (2019). Inadequate DNA damage repair promotes mammary transdifferentiation, leading to BRCA1 breast cancer. Cell.

[16] van Roy F., Berx G. (2008). The cell-cell adhesion molecule E-cadherin. Cell Mol. Life Sci..

[17] Corso G., Figueiredo J., De Angelis S.P., Corso F., Girardi A., Pereira J. (2020). E-cadherin deregulation in breast cancer. J. Cell Mol. Med..

[18] Padmanaban V., Krol I., Suhail Y., Szczerba B.M., Aceto N., Bader J.S. (2019). E-cadherin is required for metastasis in multiple models of breast cancer. Nature.

[19] Bai F., Zhang L.H., Liu X., Wang C., Zheng C., Sun J. (2021). GATA3 functions downstream of BRCA1 to suppress EMT in breast cancer. Theranostics.

[20] Batlle E., Sancho E., Franci C., Dominguez D., Monfar M., Baulida J. (2000). The transcription factor snail is a repressor of E-cadherin gene expression in epithelial tumour cells. Nat. Cell Biol..

[21] Bolos V., Peinado H., Perez-Moreno M.A., Fraga M.F., Esteller M., Cano A. (2003). The transcription factor Slug represses E-cadherin expression and induces epithelial to mesenchymal transitions: a comparison with Snail and E47 repressors. J. Cell Sci..

[22] Vesuna F., van Diest P., Chen J.H., Raman V. (2008). Twist is a transcriptional repressor of E-cadherin gene expression in breast cancer. Biochem. Biophys. Res. Commun..

[23] Yan W., Cao Q.J., Arenas R.B., Bentley B., Shao R. (2010). GATA3 inhibits breast cancer metastasis through the reversal of epithelial-mesenchymal transition. J. Biol. Chem..

[24] Fujita Y., Krause G., Scheffner M., Zechner D., Leddy H.E., Behrens J. (2002). Hakai, a c-Cbl-like protein, ubiquitinates and induces endocytosis of the E-cadherin complex. Nat. Cell Biol..

[25] Yang J.Y., Zong C.S., Xia W., Wei Y., Ali-Seyed M., Li Z. (2006). MDM2 promotes cell motility and invasiveness by regulating E-cadherin degradation. Mol. Cell Biol..

[26] Atanassov B.S., Koutelou E., Dent S.Y. (2011). The role of deubiquitinating enzymes in chromatin regulation. FEBS Lett..

[27] Ting X., Xia L., Yang J., He L., Si W., Shang Y. (2019). USP11 acts as a histone deubiquitinase functioning in chromatin reorganization during DNA repair. Nucleic Acids Res..

[28] Wiltshire T.D., Lovejoy C.A., Wang T., Xia F., O'Connor M.J., Cortez D. (2010). Sensitivity to poly(ADP-ribose) polymerase (PARP) inhibition identifies ubiquitin-specific peptidase 11 (USP11) as a regulator of DNA double-strand break repair. J. Biol. Chem..

[29] Liao Y., Zhou D., Wang P., Yang M., Jiang N. (2022). Ubiquitin specific peptidase 11 as a novel therapeutic target for cancer management. Cell Death Discov..

[30] Guo T., Tang H., Yuan Z., Zhang E., Wang X. (2022). The dual role of USP11 in cancer. J. Oncol..

[31] Park M.K., Yao Y., Xia W., Setijono S.R., Kim J.H., Vila I.K. (2019). PTEN self-regulates through USP11 via the PI3K-FOXO pathway to stabilize tumor suppression. Nat. Commun..

[32] Chiang S.Y., Wu H.C., Lin S.Y., Chen H.Y., Wang C.F., Yeh N.H. (2021). Usp11 controls cortical neurogenesis and neuronal migration through Sox11 stabilization. Sci. Adv..

[33] Yan Y., Wang X., Chaput D., Shin M.K., Koh Y., Gan L. (2022). X-linked ubiquitin-specific peptidase 11 increases tauopathy vulnerability in women. Cell.

[34] Orthwein A., Noordermeer S.M., Wilson M.D., Landry S., Enchev R.I., Sherker A. (2015). A mechanism for the suppression of homologous recombination in G1 cells. Nature.

[35] Herold S., Kalb J., Buchel G., Ade C.P., Baluapuri A., Xu J. (2019). Recruitment of BRCA1 limits MYCN-driven accumulation of stalled RNA polymerase. Nature.

[36] Shamir E.R., Ewald A.J. (2015). Adhesion in mammary development: novel roles for E-cadherin in individual and collective cell migration. Curr. Top Dev. Biol..

[37] Phillips S., Kuperwasser C. (2014). SLUG: critical regulator of epithelial cell identity in breast development and cancer. Cell Adh. Migr..

[38] Stadler M., Scherzer M., Walter S., Holzner S., Pudelko K., Riedl A. (2018). Exclusion from spheroid formation identifies loss of essential cell-cell adhesion molecules in colon cancer cells. Sci. Rep..

[39] He J., Wang F., Luo F., Chen X., Liang X., Jiang W. (2017). Effects of long term low- and high-dose sodium arsenite exposure in human transitional cells. Am. J. Transl. Res..

[40] Ribeiro A.S., Sousa B., Carreto L., Mendes N., Nobre A.R., Ricardo S. (2013). P-cadherin functional role is dependent on E-cadherin cellular context: a proof of concept using the breast cancer model. J. Pathol..

[41] Tinkle C.L., Lechler T., Pasolli H.A., Fuchs E. (2004). Conditional targeting of E-cadherin in skin: insights into hyperproliferative and degenerative responses. Proc. Natl. Acad. Sci. U. S. A..

[42] Ishiyama N., Lee S.H., Liu S., Li G.Y., Smith M.J., Reichardt L.F. (2010). Dynamic and static interactions between p120 catenin and E-cadherin regulate the stability of cell-cell adhesion. Cell.

[43] Nino C.A., Sala S., Polo S. (2019). When ubiquitin meets E-cadherin: plasticity of the epithelial cellular barrier. Semin. Cell Dev. Biol..

[44] Hartsock A., Nelson W.J. (2012). Competitive regulation of E-cadherin juxtamembrane domain degradation by p120-catenin binding and Hakai-mediated ubiquitination. PLoS One.

[45] Baranwal S., Alahari S.K. (2009). Molecular mechanisms controlling E-cadherin expression in breast cancer. Biochem. Biophys. Res. Commun..

[46] Chao Y.L., Shepard C.R., Wells A. (2010). Breast carcinoma cells re-express E-cadherin during mesenchymal to epithelial reverting transition. Mol. Cancer.

[47] Wong S.H.M., Fang C.M., Chuah L.H., Leong C.O., Ngai S.C. (2018). E-cadherin: its dysregulation in carcinogenesis and clinical implications. Crit. Rev. Oncol. Hematol..

[48] Goulding H., Pinder S., Cannon P., Pearson D., Nicholson R., Snead D. (1995). A new immunohistochemical antibody for the assessment of estrogen receptor status on routine formalin-fixed tissue samples. Hum. Pathol..

[49] Qian T., Liu C., Ding Y., Guo C., Cai R., Wang X. (2020). PINCH-1 interacts with myoferlin to promote breast cancer progression and metastasis. Oncogene.

[50] Zhu X., Xue J., Jiang X., Gong Y., Gao C., Cao T. (2022). TRIM21 suppresses CHK1 activation by preferentially targeting CLASPIN for K63-linked ubiquitination. Nucleic Acids Res..

[51] Bai F., Liu S., Liu X., Hollern D.P., Scott A., Wang C. (2021). PDGFRbeta is an essential therapeutic target for BRCA1-deficient mammary tumors. Breast Cancer Res..

