# Supplementary material for: USP11 deubiquitinates E-cadherin and maintains the luminal fate of mammary tumor cells to suppress breast cancer
Source: J Biol Chem. 2024 Sep 11;300(10):107768. doi: 10.1016/j.jbc.2024.107768 (PMC11497446; doi:10.1016/j.jbc.2024.107768)
Supplement: Supplementary Figures S1–S6 [file mmc1.pdf]

Supplymentary Data
Figure. S1

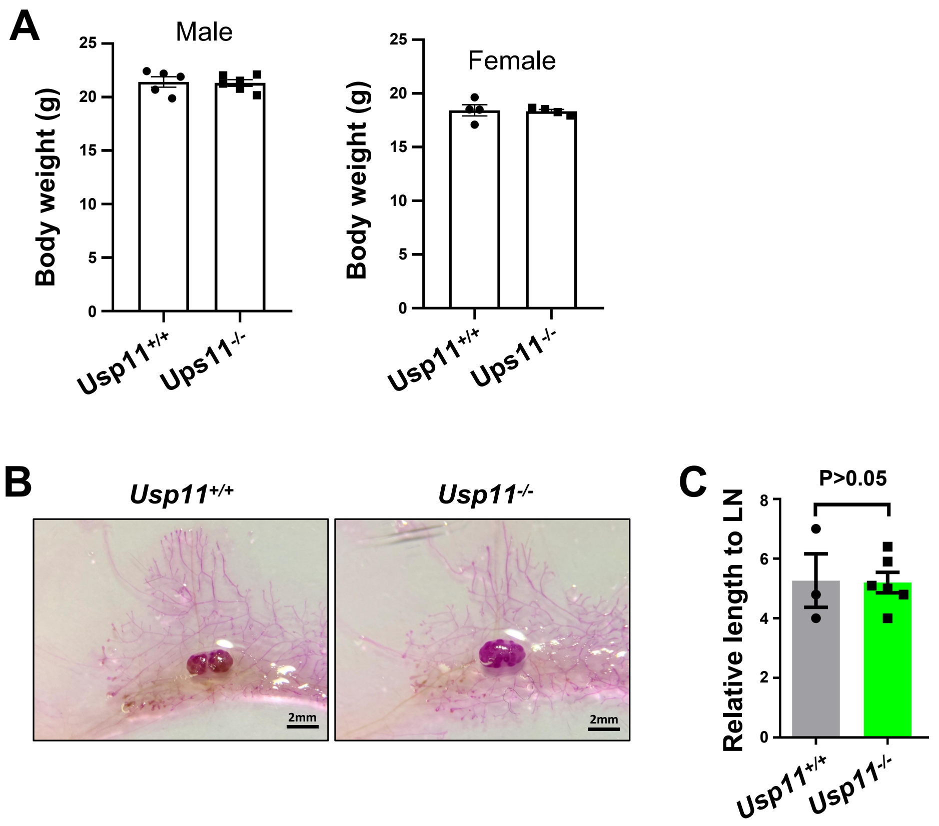

Figure S1. Characterization of Usp11 knock-out mice

(A) Quantification of body weight from 8-week-old male (left) and female (right) mice. The results represent the mean ± SEM. N = 4 for male *Usp11*$^{+/+}$ mice; N = 4 for male *Usp11*$^{-/-}$ mice; N = 5 for female *Usp11*$^{+/+}$ mice; N = 6 for female *Usp11*$^{-/-}$ mice. (B) Whole mount staining of mammary glands from 6-week-old *Usp11*$^{+/+}$ and *Usp11*$^{-/-}$ mice. (C) Quantification of the mammary tube infiltrating length from (B). The infiltrating length is calculated from the furthest front edge of infiltration to the center of the lymph node. The results represent the mean ± SEM of three *Usp11*$^{+/+}$ and six *Usp11*$^{-/-}$ mice.

Supplymentary Data
Figure. S2

A

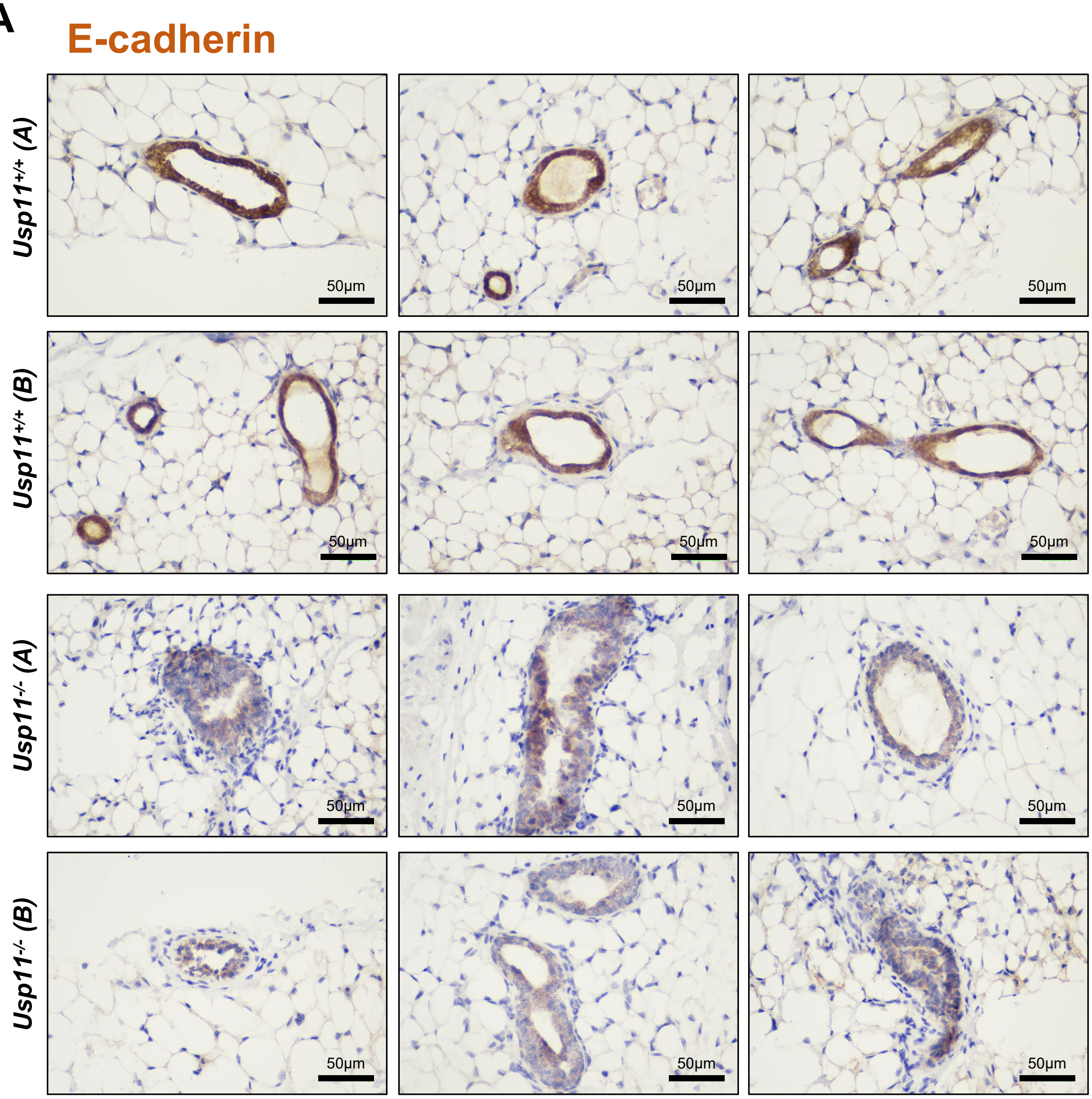

Supplymentary Data
Figure. S2

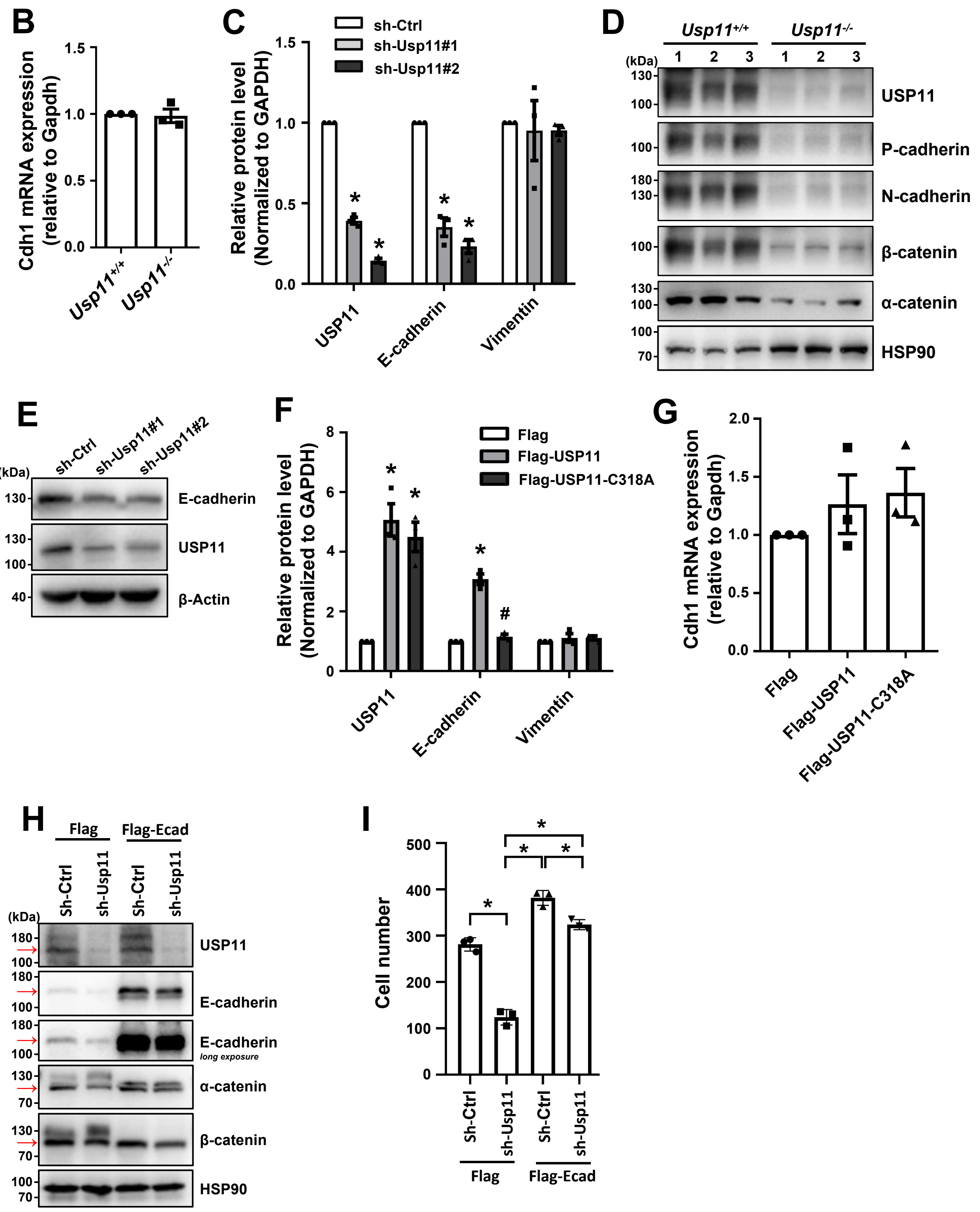

Supplymentary Data
Figure. S2

Figure S2. Analysis of Usp11-deficient mammary glands and cells

(A) Representative IHC staining of E-cadherin in mammary glands from 5-week-old *Usp11*$^{+/+}$ and *Usp11*$^{-/-}$ female mice. (B) qRT-PCR analysis of mRNA expression of Cdh1 in *Usp11*$^{+/+}$ and *Usp11*$^{-/-}$ MECs. Gapdh was used as an internal control. The results represent the mean ± SEM of three individual samples. (C) Quantification of the USP11, E-cadherin, and Vimentin levels in (Figure 2G). The results represent the mean ± SEM of three individual samples. *P<0.05 vs sh-Control (sh-Ctrl) group. (D) Western blotting analysis of the mammary glands from 5-week-old *Usp11*$^{+/+}$ and *Usp11*$^{-/-}$ mice. HSP90 was used as an internal control. N=3 for each group. (E) Western blotting analysis of the protein expression of E-cadherin and USP11 in sh-Ctrl, sh-Usp11#1, or sh-Usp11#2 lentivirus-infected HC11 cells. β-Actin was used as an internal control. (F) Quantification of the USP11, E-cadherin, and Vimentin levels in (Figure 2H). The results represent the mean ± SEM of three individual samples. *P<0.05 vs Flag group; #P<0.01 vs Flag-USP11 group. (G) qRT-PCR analysis of Cdh1 in mouse mammary tumor cells infected with Flag, Flag-USP11, and Flag-USP11-C318A. Gapdh was used as an internal control. The results represent the mean ± SEM of three individual samples. (H, I) The USP11 stable knockdown (sh-Usp11) and control (sh-Ctrl) primary mouse *MMTV-PyMT* mammary tumor cells were transiently transfected with Flag-E-cadherin and Flag plasmids, respectively. The expression of the genes indicated was detected by western blotting (H). 100,000 cells per well were re-seeded in 12-well plate, cultured for 12 hours, and washed. The adhered cells in the plate were counted and statistically analyzed (I). N=4, *P<0.05 was considered significant.

Supplymentary Data
Figure. S3

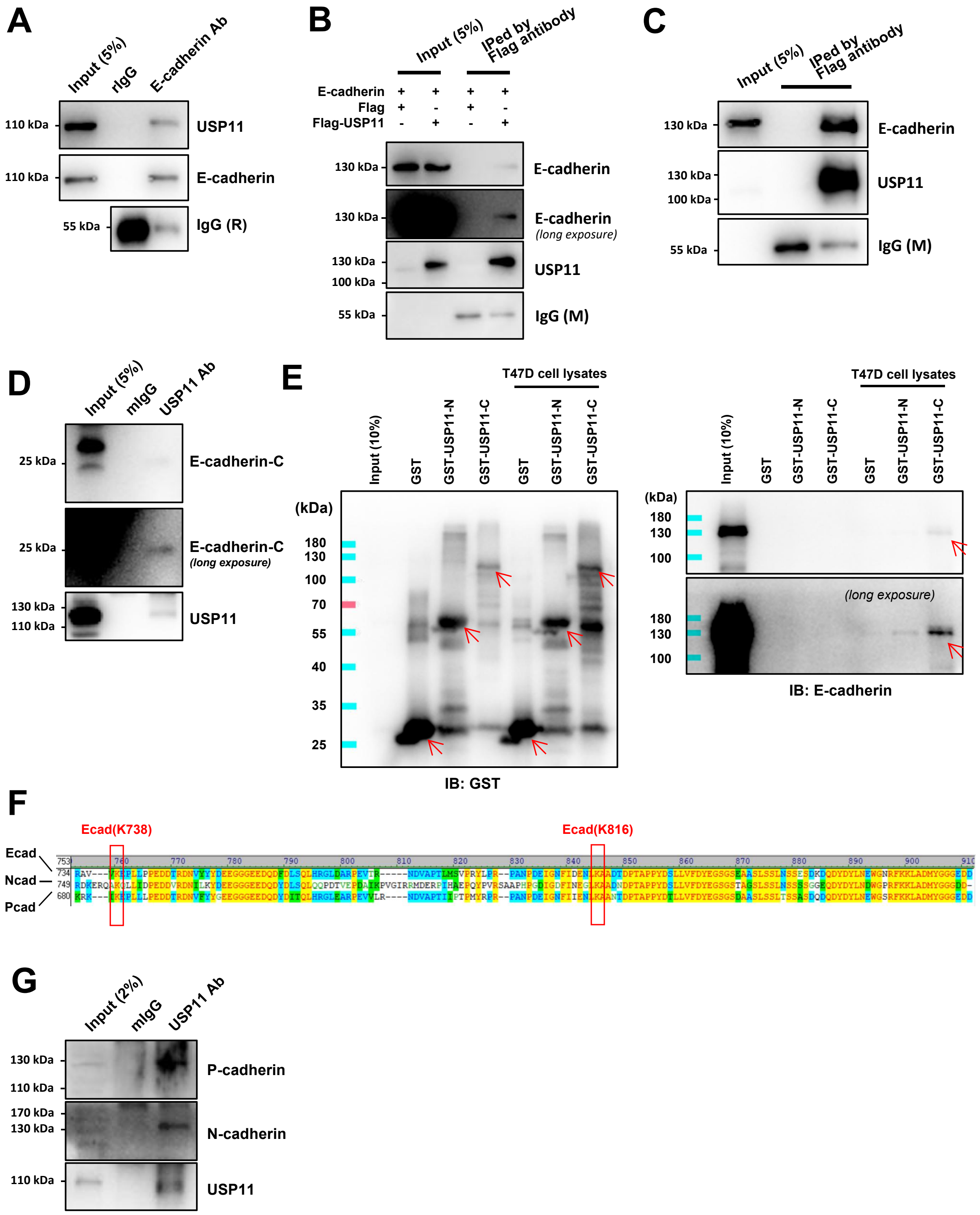

Supplymentary Data
Figure. S3

Figure S3. The interaction of Usp11 and E-cadherin

(A) Immunoprecipitations were performed in mouse mammary tumor cell lysates using anti-E-cadherin antibody or IgG. The expression of USP11 and E-cadherin in input and immunoprecipitated samples was detected by western blotting. (B) HEK293T cells were transfected with Flag or Flag-USP11 for 48 hours, then immunoprecipitations were performed in the mixture of HEK293T and T47D cell lysates using anti-Flag antibody. T47D cells were used to provide external E-cadherin protein in immunoprecipitation reactions. The expression of E-cadherin and USP11 in input and immunoprecipitated samples was detected by western blotting. (C) Human T47D cells were transfected with Flag or Flag-USP11 for 48 hours, then immunoprecipitations were performed in these cell lysates using anti-Flag antibody. The expression of E-cadherin and USP11 in input and immunoprecipitated samples was detected by western blotting. (D) HEK293T cells were transfected with Flag-E-cadherin-C (731-882aa) and Flag-USP11 for 48 hours. Immunoprecipitations were performed in these cell lysates using anti-USP11 antibody or IgG. The expression of E-cadherin-C and USP11 in input and immunoprecipitated samples was detected by western blotting. (E) Purified GST or GST-tagged N-terminal and C-terminal fragments of USP11 were used to interact with E-cadherin in T47D cell lysates, and the eluted proteins were analyzed by western blotting using GST or E-cadherin primary antibody. (F) The similarity of human E-cadherin (Ecad), N-cadherin (Ncad), and P-cadherin (Pcad) was analyzed by Vector NTI Suite 8. Alignment of cytoplasmic domains was shown. Please note, the K738 and K816 ubiquitin sites of E-cadherin were conserved in P-cadherin and N-cadherin according to the alignment result. (G) Immunoprecipitations were performed in T47D cell lysates using anti-USP11 antibody or IgG. The expression of P-cadherin, N-cadherin, and USP11 in input and immunoprecipitated samples was detected by western blotting.

Supplymentary Data
Figure. S4

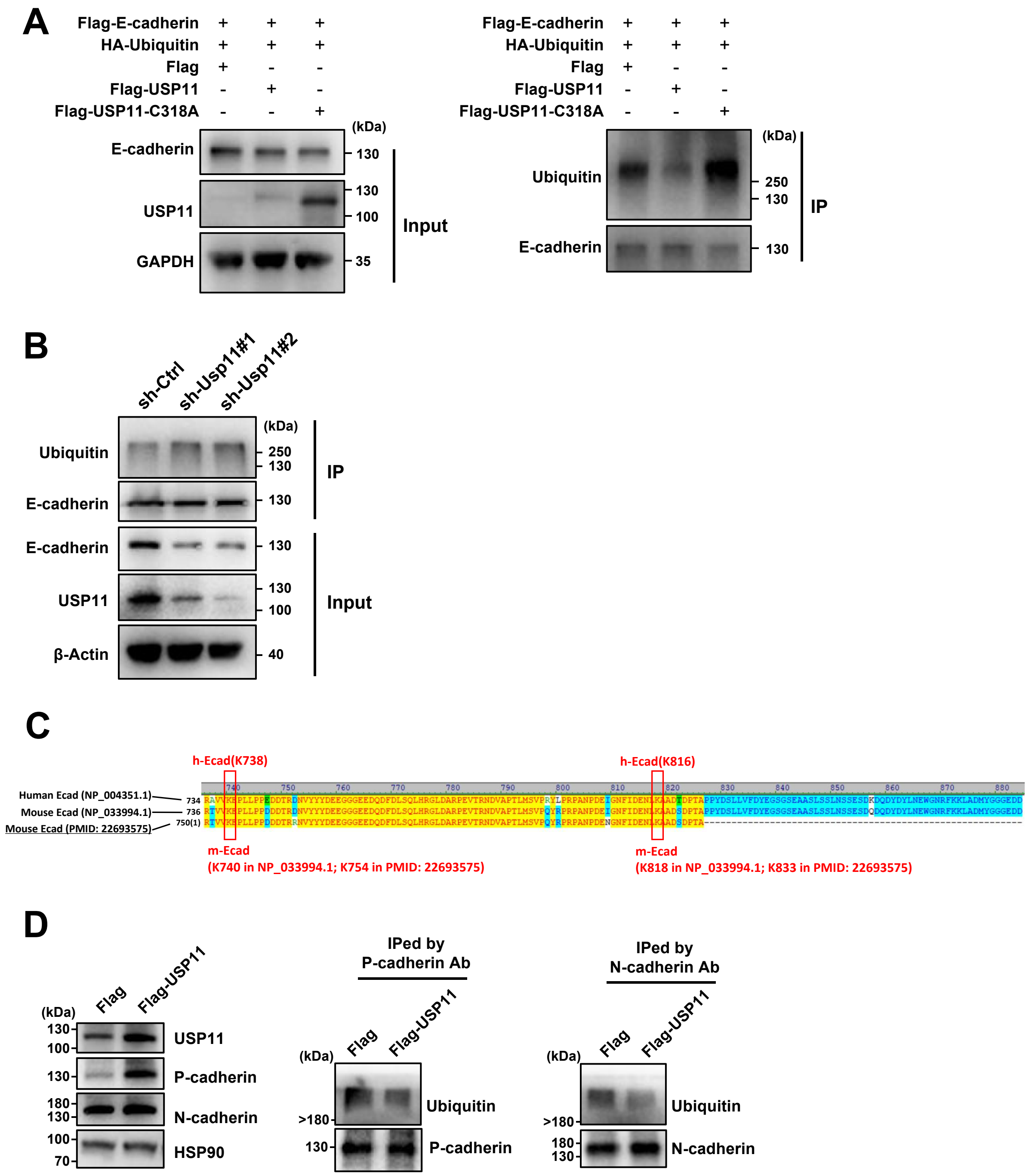

Supplymentary Data
Figure. S4

Figure S4. Deubituitination of E-cadherin by USP11

(A) HEK293T cells were transfected with Flag, Flag-USP11, or Flag-USP11-C318A plasmids along with Flag-E-cadherin and HA-Ubiquitin overexpression plasmids for 48 hours, then immunoprecipitations were performed in these cell lysates using anti-E-cadherin antibody or IgG. The expression of E-cadherin and USP11 in input samples (left) and the expression of ubiquitin and E-cadherin in immunoprecipitated samples (right) were detected by western blotting. GAPDH was used as an internal control for input samples. (B) Murine luminal tumor cells infected with sh-Usp11#1, sh-Usp11#2, or sh-Ctrl lentivirus for 48 hours were treated with MG132 (20 nM) for 6 hours and then lysed. Immunoprecipitation was performed using E-cadherin primary rabbit polyclonal antibody in the cell lysates. Ubiquitin and E-cadherin in immunoprecipitates, and Ubiquitin, E-cadherin, and USP11 in input samples were detected by Western blotting. β-Actin was used as an internal control for input samples. (C) The similarity of human (upper sequence) and mouse (middle and lower sequence) E-cadherin was analyzed by Vector NTI Suite 8. Alignment of the cytoplasmic domain of human E-cadherin (743-882aa, NP_004351.1), mouse E-cadherin (736-884aa, NP_033994.1), and mouse E-cadherin (750-841aa, as described in PMID: 22693575) was shown. Please note, the ubiquitin sites K740 and K818 in mouse E-cadherin were conserved as K738 and K816 in human E-cadherin. (D) T47D cells infected with Flag or Flag-USP11 were treated with MG132 for 6 hours and then lysed. Immunoprecipitation was taken using P-cadherin or N-cadherin antibody in the cell lysates. Ubiquitin, P-cadherin, and N-cadherin in immunoprecipitates, and P-cadherin, N-cadherin, USP11, and HSP90 in input samples were detected by Western blotting.

Supplymentary Data
Figure. S5

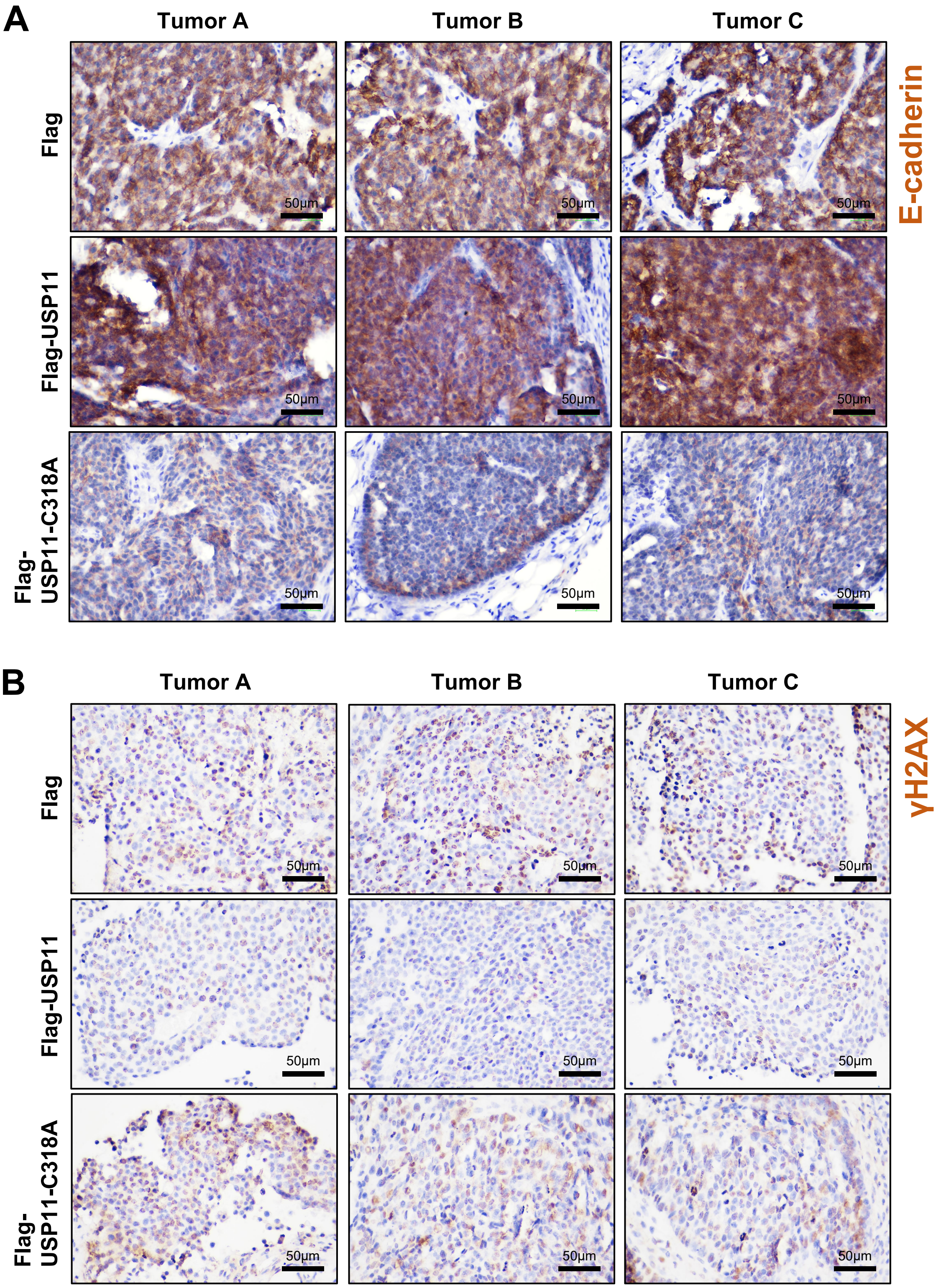

Supplymentary Data
Figure. S5

Figure. S5. IHC analysis of the gene expression in xenograft tumors.

(A, B) Tumors generated by mouse mammary tumor cells stably expressing Flag, Flag-USP11, or Flag-USP11-C318A were analyzed with antibodies against E-cadherin (A) and γH2AX (B). Representative IHC staining results from three individual tumors are shown.

Supplymentary Data
Figure. S6

A Breast tumor_01_GS2021025293

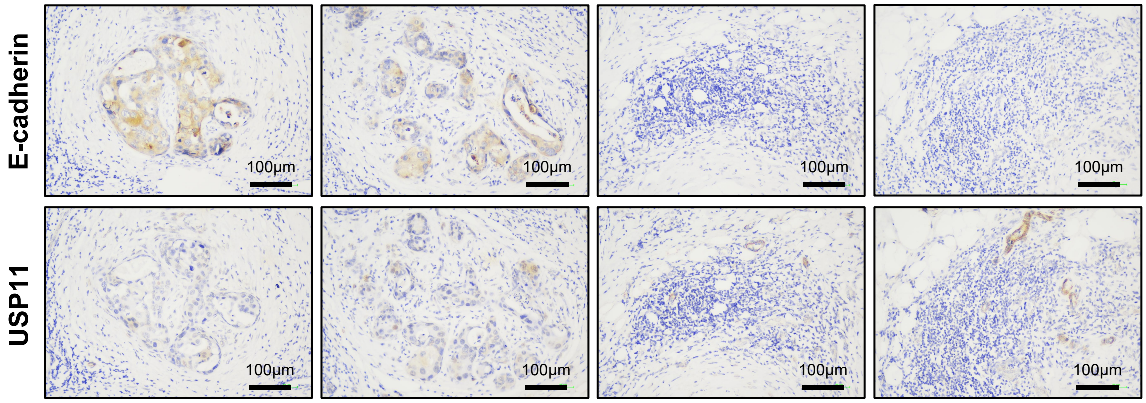

B Breast tumor_02_GS2021026422

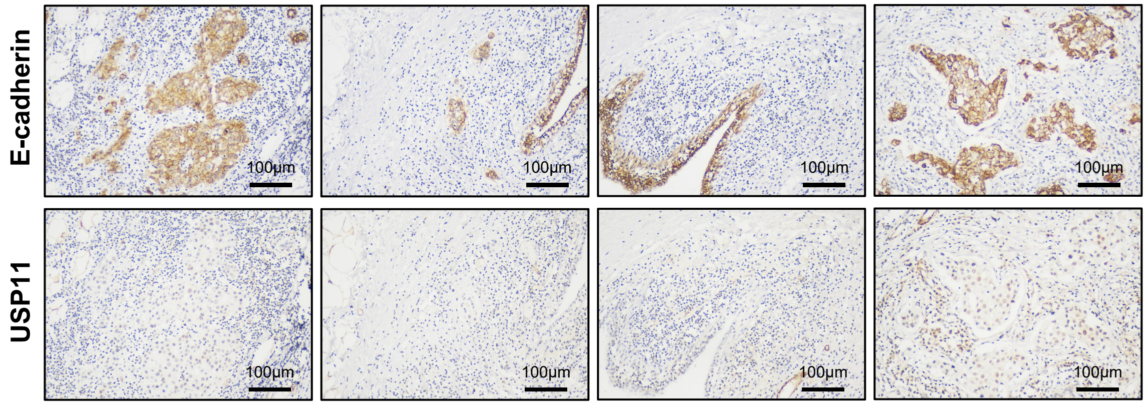

C Breast tumor_03_GS2022003698

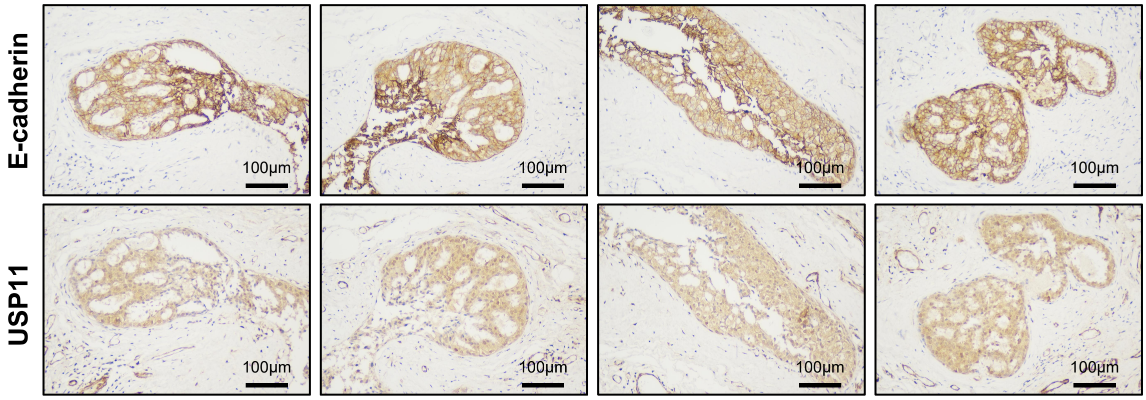

Supplymentary Data
Figure. S6

D **Breast tumor_04_GS2022003573**

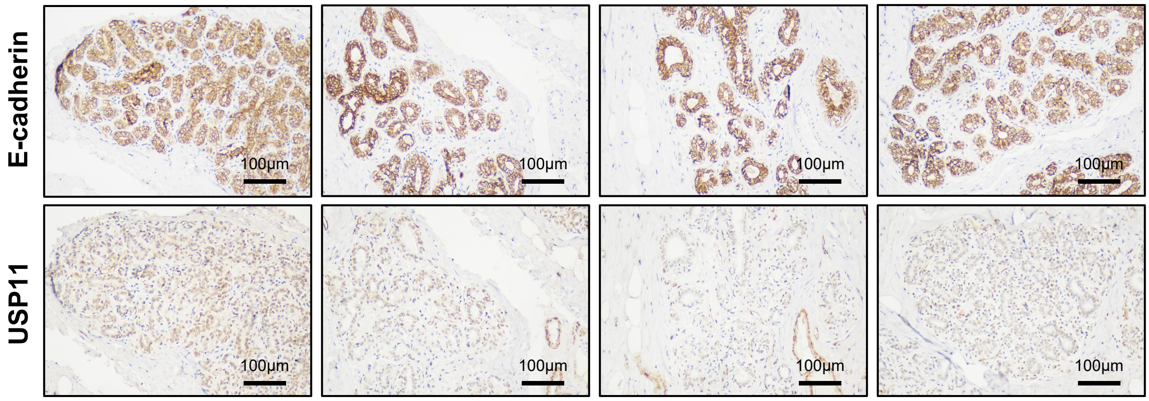

E **Breast tumor_05_GS2022000680**

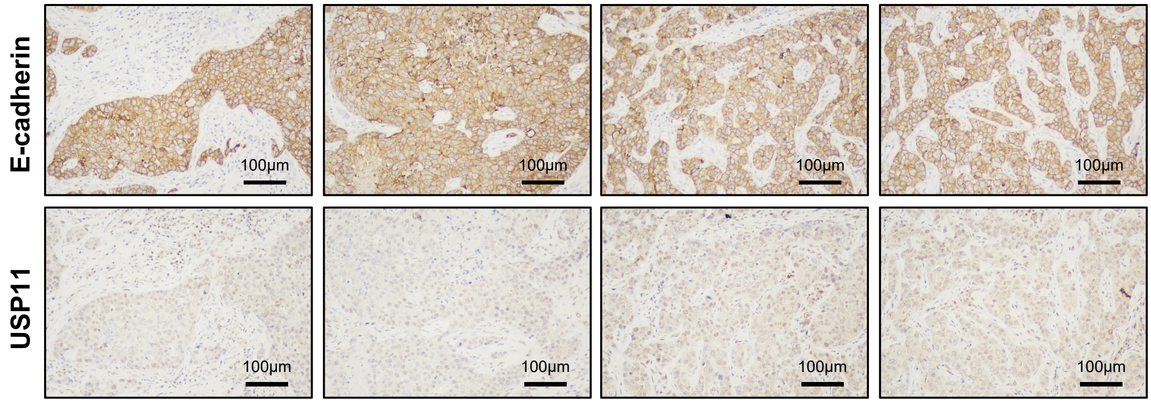

F **Breast tumor_06_GS2022015834**

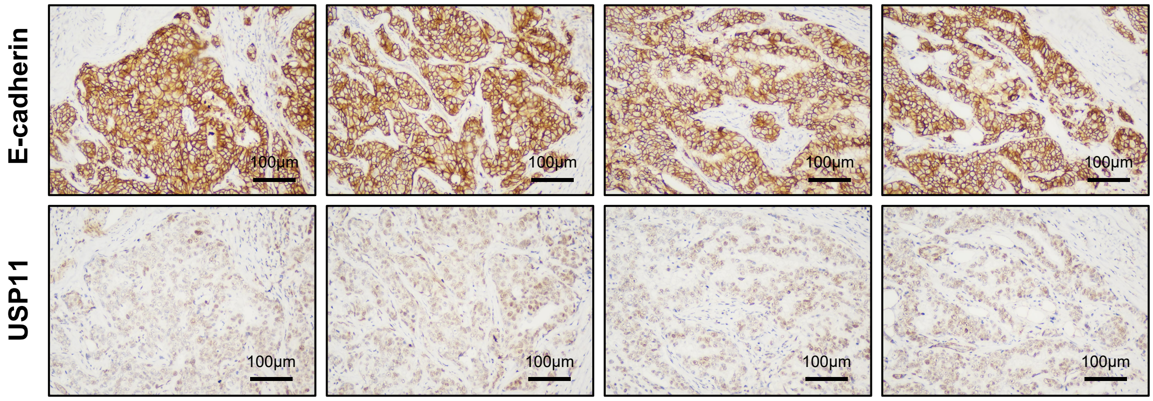

Supplymentary Data
Figure. S6

G **Breast tumor_07_GS2022002118**

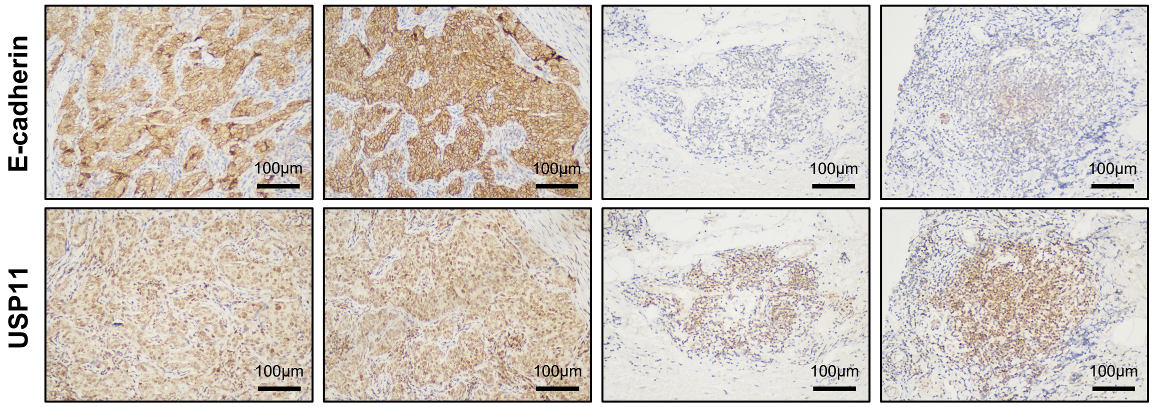

H **Breast tumor_08_GS2022002113**

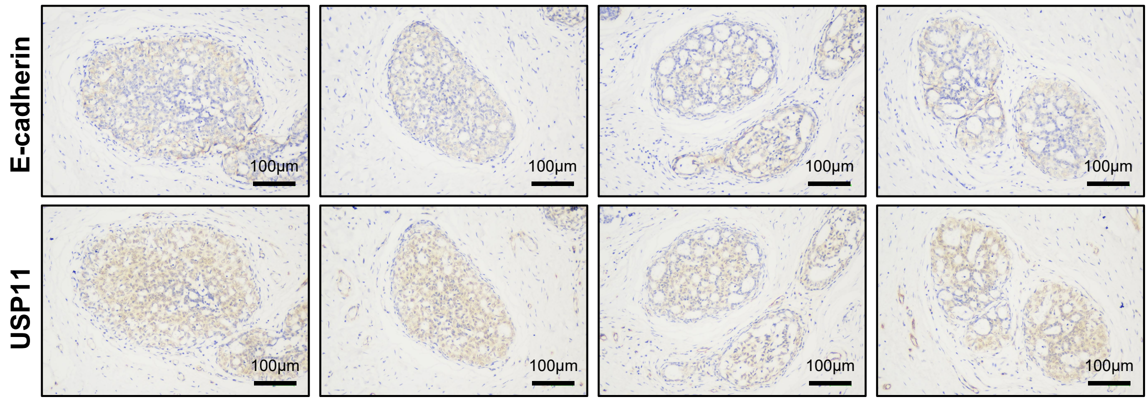

I **Breast tumor_09_GS2022001812**

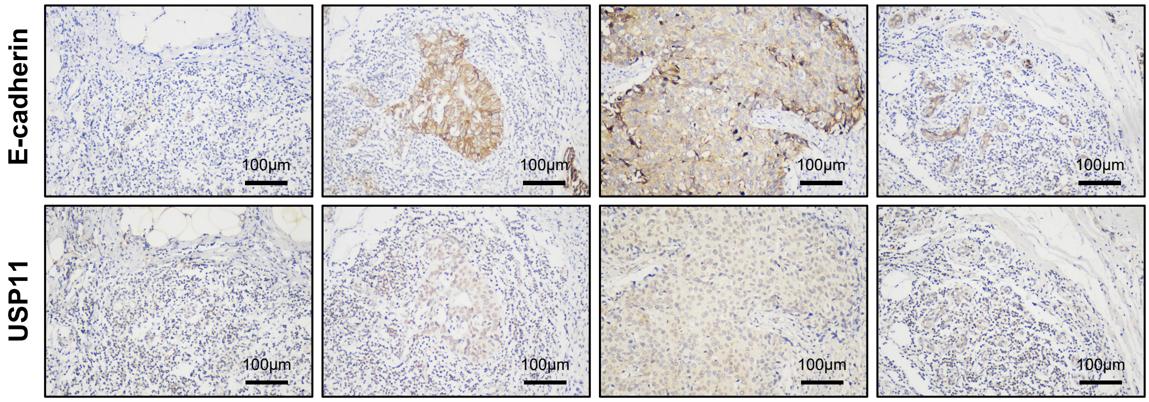

Supplymentary Data
Figure. S6

J

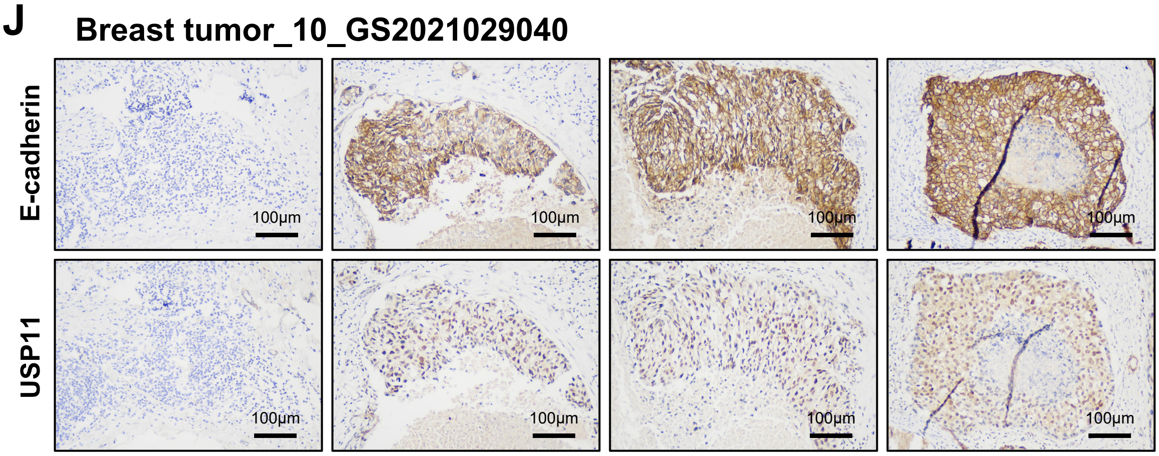

Figure S6. IHC analysis of human breast cancer samples.

(A-J) Representative IHC staining of E-cadherin and USP11 in human breast cancers. Representative results from four individual sub-tumor nodules/foci of each tumor sample are shown.
